# Isolation, Characterization, and Anticancer Evaluation of Alkaloids from *Eumachia montana* (Rubiaceae)

**DOI:** 10.1021/acsomega.5c02401

**Published:** 2025-08-04

**Authors:** Yuye Shuai, Dong-Hyun Kim, Kuan-Hon Lim, Premanand Krishnan, Yun-Yee Low, Kien-Thai Yong, Tracey D. Bradshaw

**Affiliations:** † School of Pharmacy, Biodiscovery Institute, 6123 The University of Nottingham, University Park, Nottingham, Nottinghamshire NG7 2RD, U.K.; ‡ School of Pharmacy, Faculty of Science and Engineering, 69861 University of Nottingham Malaysia, Semenyih, Selangor 43500, Malaysia; § Department of Chemistry, Faculty of Science, 37447 Universiti Malaya, Kuala Lumpur 50603, Malaysia; ∥ Institute of Biological Sciences, Faculty of Science, 37447 Universiti Malaya, Kuala Lumpur 50603, Malaysia

## Abstract

The dimeric (−)-calycanthine (**1**), the hexameric (+)-oleoidine (**2**), the heptameric (+)-caledonine (**3**), the octameric (+)-eumatanine (**4**), and a tricyclic pyrroloquinoline alkaloid, eumatricine (**5**), are alkaloids isolated from the leaves of *Eumachia montana*; compounds **4** and **5** are novel, previously uncharacterized alkaloids. Preliminary anticancer assays, including MTT, cell count, and clonogenic assays, revealed potent inhibition of cell growth and colony formation by compounds **2–4** against seven carcinoma cell lines (including breast, colorectal, lung, and glioblastoma multiforme cell lines). The GI_50_ values of compounds **2–4** ranged from 0.1 to 2.7 μM, and potent cytotoxic activity against HCT-116 and MCF-7 was observed (**3** > **4** > **2**). Cell cycle analysis and apoptosis assays revealed that compound **4** evokes profound apoptosis without cell cycle phase-specific arrest, accompanied by caspase activation, corroborating apoptosis-induction. Detection of ROS induced by **4** infers a role for oxidative stress in anticancer activity. γH2AX detection indicates the presence of DNA double-strand breaks in cells treated with compound **4**. Visualization of **4**-treated cells by fluorescence microscopy implies multiple cell death pathways are simultaneously triggered (e.g., apoptosis, autophagy, necrosis, and paraptosis).

## Graphical abstract

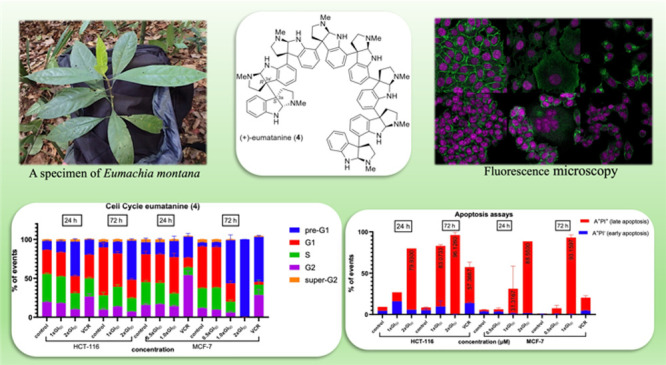

## Introduction

Evolutionary fine-tuned and possessing vast structural diversity and complexity, natural products (NPs) offer an invaluable source of therapeutic agents. Specifically in the anticancer arena, NPs provide molecular scaffolds for drug discovery. Oligocyclotryptamine alkaloids (also termed polypyrroloindoline alkaloids) represent a fascinating family of NPs known for their diverse therapeutic bioactivities, including anticancer (quadrigemine H, quadrigemine C, psychotridine, isopsychotridine C from *Eumachia forsteriana*), analgesic ((+)-chimonanthine, hodgkinsine, quadrigemine C, psychotridine from *Palicourea colorata*), antimicrobial (psychotrimine from *Eumachia rostrata*), and antithrombotic (Psm2 from *Selaginella moellendorffii*) effects.^1–10^ such properties validate the search for novel alkaloids in uncharted *Eumachia* species such as—*Eumachia montana* (Blume) I.M.Turner (syn. *Psychotria montana*). To the best of our knowledge, *Eumachia montana* has not previously been studied phytochemically or biologically according to the Web of Science and Reaxys. However, traditionally, the roots of *E. montana* are applied externally for poulticing ulcers and swellings, or have been made into a decoction that is consumed orally to treat constipation.^11,12^

The genus *Eumachia* and its relative *Palicourea* are well-known for their cyclotryptamine constituent, but only nine species from the genus *Eumachia* have been phytochemically investigated so far. The isolation of cyclotryptamines has been reported from the Amazon plants *Palicourea colorata* and *Palicourea muscosa*, as well as the South Pacific ocean’s plants *Eumachia forsteriana*, *Eumachia lyciiflora*, and *Eumachia oleoides*, namely, (+)-chimonanthine, hodgkinsine, quadrigemine H, quadrigemine C, psychotridine, isopsychotridine C, oleoidine (**2**), and caledonine (**3**) (Figure 1).^13–15^ Additionally, complex polypyrroloindoline alkaloids (containing four or more indole units) quadrigemine H, quadrigemine C, and psychotridine have been isolated from *Eumachia forsteriana*, *Palicourea colorata*, and *Eumachia oleoides*, while compounds **2** and **3** have only been isolated from *Eumachia oleoides*.^1,13,15,16^ It is noteworthy that compounds **2** and **3** have also been isolated from the leaves of *E. montana* in this study, along with **4** as major alkaloidal components.

**Figure 1:**
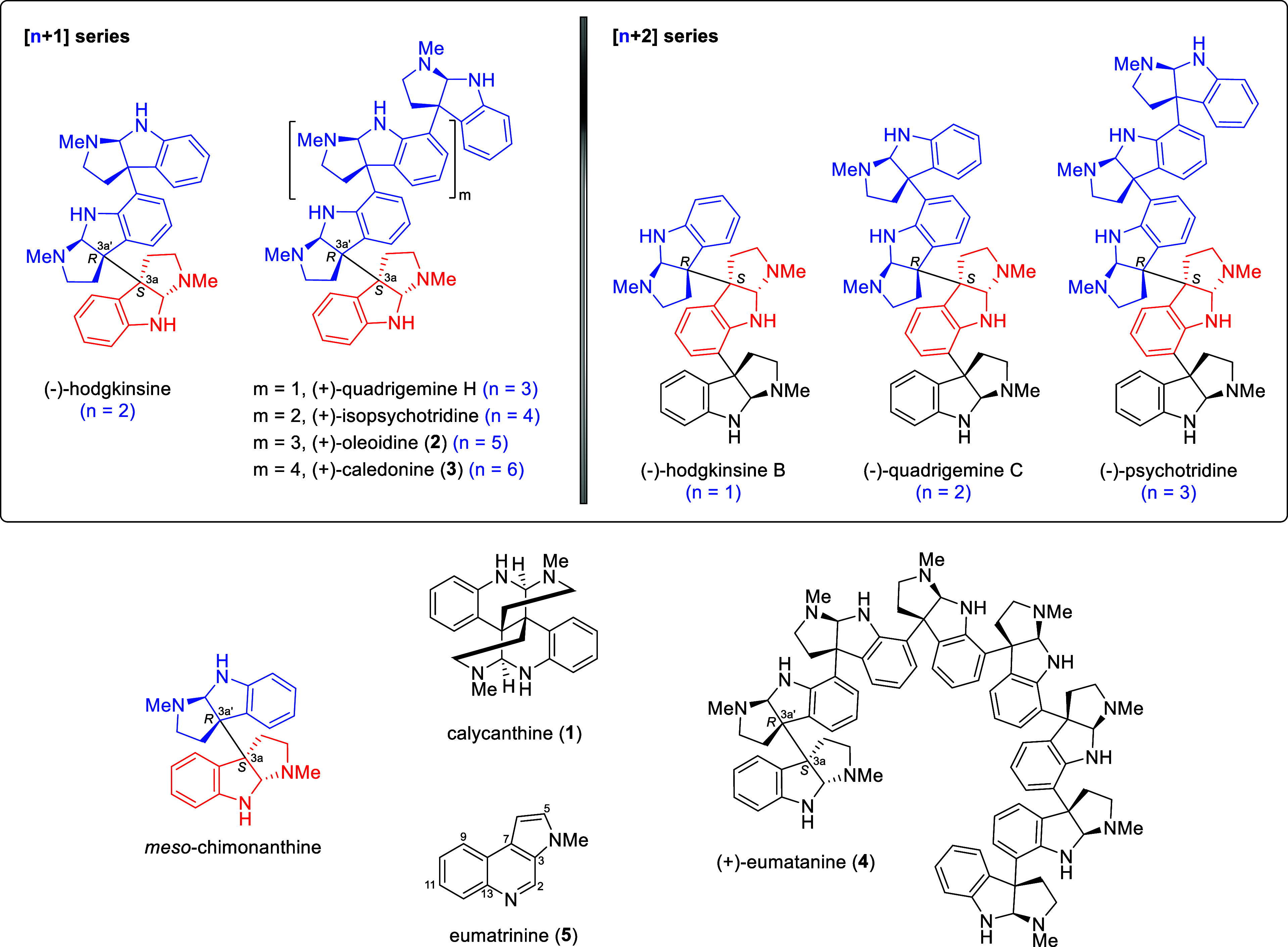
Representative [*n*+1] and [*n*+2] series of oligocyclotryptamine alkaloids and structures of alkaloids 1–5.

These alkaloids are derived from the basic building block of *cis*-pyrrolidino­[2,3-*b*]­indoline (also referred to as cyclotryptamine), and their structure typically features the dimeric 3*a*-3*a*′-bispyrrolidino­[2,3-*b*]­indoline core unit.^17^ Higher-order oligomers in this family are primarily formed by attaching additional 3*a* *R*-*cis*-pyrrolidino­[2,3-*b*]­indoline units at the benzylic carbon (position-3a) to the aromatic carbon (position-7) of the preceding structure. *meso*-Chimonanthine often serves as the core structure in these alkaloids (Figure 1). These oligocyclotryptamine alkaloids, particularly those based on the *meso*-chimonanthine core, can be further categorized into the [*n* + 1] and [*n* + 2] series.^13,18^ Representative alkaloids of these series are illustrated in Figure 1.

The structural and stereochemical characterization of some higher-order oligocyclotryptamines has previously been complicated by atropisomerism, leading to signal broadening in NMR spectra. However, mass spectrometry has been invaluable, as the characteristic fragmentation of the C3a–C3a′ σ bond provides crucial insights into the positioning of this linkage within each oligomer.^13,19^ Recent advancements in chemical synthesis, which enable stereocontrolled coupling of cyclotryptamine monomers, have helped overcome longstanding challenges in determining and validating the structures and stereochemistry of these complex alkaloids, including (−)-hodgkinsine, (+)-quadrigemine H, (+)-isopsychotridine, (+)-oleoidine, (+)-caledonine, (−)-hodgkinsine B, (−)-quadrigemine C, and (−)-psychotidine (Figure 1).^17,18^

Our interest in *Eumachia montana* arose from the identification of potent cytotoxic activity in its leaf alkaloid extract against a panel of cancer cell lines. Chromatographic isolation of this extract yielded five alkaloids: the dimeric (−)-calycanthine (**1**), the hexameric (+)-oleoidine (**2**), the heptameric (+)-caledonine (**3**), the octameric (+)-eumatanine (**4**), and a tricyclic pyrroloquinoline alkaloid, eumatricine (**5**). Of these, compounds **4** and **5** are previously undescribed. Herein, we report the isolation and structure determination of these alkaloids, along with preliminary anticancer cytotoxic data for compounds **2**–**4** (e.g., MTT and clonogenic assays) and the discovery of cellular mechanisms of action of compounds **3** and **4** (e.g., cell cycle and apoptosis assays).

## Results and Discussion

### Structure Elucidation

While compound **1** ([α]_D_ −585 (*c* 0.53, MeOH)) was readily identified as (−)-calycanthine through X-ray diffraction and ^1^H NMR spectroscopy (Figures S1 and S2), structure determination for compounds **2** and **3** proved significantly more challenging. The 1D and 2D NMR spectra obtained for compounds **2** and **3** were unsatisfactory, exhibiting complex and unresolved signals even at low temperatures (−30 °C) (Figures S3–S11). However, high-resolution electrospray ionization mass spectrometry (HRESIMS) data revealed that they are hexameric and heptameric cyclotryptamine alkaloids, respectively (Figure S12). Additionally, HRESIMS data for compound **2** exhibited a [M + H]^+^ peak at *m*/*z* 1035.6217 (C_66_H_74_N_12_ + H^+^), accompanied by [M + 2H]^2+^ and [M + 3H]^3+^ peaks at *m*/*z* 518.3155 (C_66_H_74_N_12_ + 2H^+^) and 345.8791 (C_66_H_74_N_12_ + 3H^+^), respectively. Similarly, compound **3** displayed a [M + H]^+^ peak at *m*/*z* 1207.7219 (C_77_H_86_N_14_ + H^+^), with corresponding [M + 2H]^2+^ and [M + 3H]^3+^ peaks at *m*/*z* 604.3649 (C_77_H_86_N_14_ + 2H^+^) and 403.2457 (C_77_H_86_N_14_ + 3H^+^). Furthermore, the [MH – 172]^+^ peaks observed in the mass spectra of both compounds indicated fragmentation patterns characteristic of the [*n*+1] series of pyrrolidino­[2,3-*b*]­indoline alkaloids, suggesting their structures to be those of oleoidine and caledonine.^13,19^ This suggestion was supported by the fact that the electronic circular dichroism (ECD) curves of both compounds matched those reported for (−)-hodgkinsine, (+)-oleoidine, and (+)-caledonine (Figure S13). Additionally, the specific optical rotation data, as well as the ^1^H and ^13^C NMR spectra, of compounds **2** ([α]_D_ + 106 (*c* 0.43, EtOH)) and **3** ([α]_D_ + 139 (*c* 0.80, EtOH)) were broadly consistent with those reported for oleoidine and caledonine, respectively.^13,18^

Eumatanine (**4**) was isolated as a colorless oil, [α]_D_ + 122 (*c* 0.33, EtOH). The NMR spectra of eumatanine (**4**) were also unresolved, even at low temperatures, and showed a general resemblance to those of compounds **2** and **3** (Figures S14–S17). However, its HRESIMS data provided crucial insights into its structure. The mass spectrum exhibited a [M + H]^+^ peak at *m*/*z* 1379.8215 (C_88_H_98_N_16_ + H^+^), indicative of an octameric cyclotryptamine alkaloid, along with multiple charged ion peaks at *m*/*z* 690.9153 ([M + 2H]^2+^), 460.6125 ([M + 3H]^3+^), and 345.7102 ([M + 4H]^4+^) (Figure S18). Furthermore, fragment ion peaks at *m*/*z* 1207.7217 ([MH – 172]^+^) and 604.3643 ([M + 2H – 172]^+^) indicated that the terminal cyclotryptamine unit is connected to seven other units via a C3a–C3a′ σ bond, concluding that **4** has a [7 + 1] arrangement of cyclotryptamine units. According to the biosynthetic pathway proposed by Scott and Mossavaghi, these alkaloids are formed by sequentially adding 3a*R*-configured *cis*-pyrrolidino­[2,3-*b*]­indoline units to the C-7 position of a preceding structure, with *meso*-chimonanthine as the core unit.^18^ Based on this pathway, a plausible stereochemical assignment for eumatanine (**4**) can be proposed, which maintains the regio- and stereochemical integrity observed in previously characterized oligomers. This proposal suggests that **4** consists of seven *R* and one *S* cyclotryptamine units (Figure 1). The identical ECD spectrum of **4**, compared to those of compounds **2** and **3** (Figure S13), further supports this stereochemical assignment. Eumatanine (**4**) thus represents the second reported octameric cyclotryptamine alkaloid, following vatamidine, which exhibits a [6 + 2] arrangement of cyclotryptamine units.^4,18^

Eumatricine (**5**) was obtained in minute amounts as a light yellowish powder. High-resolution mass spectrometry (HRESIMS) showed a [M + H]^+^ peak at *m*/*z* 183.0932, allowing for the deduction of the molecular formula C_12_H_10_N_2_, which corresponds to nine degrees of unsaturation. The ^13^C NMR data of **5** (Table 1) showed the presence of 12 carbons, which agrees with the molecular formula established by the HRMS data. Of the 12 carbons detected, 11 were due to aromatic resonances, while one was due to an aliphatic resonance. The ^1^H NMR data of **5** (Table 1) indicated the presence of seven aromatic methines and a methyl group. The molecular formula of compound **5**, indicating the presence of 10 hydrogen atoms, suggests a predominantly unsaturated structure with the methyl group as the only aliphatic partial structure. The methyl group, resonating at δ_H_ 4.04, indicates the presence of an *N*-methyl group as the molecular formula lacks an oxygen atom. The *N*-methyl group was deduced to be part of the pyrrole moiety based on the HMBC three-bond correlations observed from CH_3_-14 to C-3 and C-5; from H-5 to C-3 and C-7; and from H-6 to C-3 (Figure 2). The distinctive methine signal observed at δ_H_ 9.04 suggests the presence of the imine function of a quinoline structure. The ^1^H NMR revealed a pair of AB doublets at δ_H_ 7.00 and 7.27 with a coupling constant of 2.9 Hz, which the COSY data attributed to a CH–CH fragment (Figure 2). Additionally, the COSY data indicated that the remaining methine signals at δ_H_ values of 8.20, 8.18, 7.60, and 7.58 correspond to a CH–CH–CH–CH partial structure, consistent with a 1,2-disubstituted benzene ring (Figure 2). The most downfield carbon resonance at δ_C_ 142.43 was attributable to the *N*-substituted aromatic C-13 based on the three-bond correlations observed from H-9 and H-11 to C-13. The presence of a quinoline structure substituted at C-3 and C-7 was supported by the three-bond correlations observed from H-2 (δ_H_ 9.04) to C-7 and C-13; from H-9 to C-11 and C-13; from H-10 to C-8 and C-12; from H-11 to C-9 and C-13; and from H-12 to C-8 and C-10. Finally, the pyrrole fragment was deduced to be fused to the quinoline structure at C-3 and C-7 based on the correlations from H-5 to C-3 and C-7, and from CH_3_-14 to C-3 and C-5. The proposed structure of eumatricine (**5**) is consistent with the full HMBC data. Additionally, the proposed structure was corroborated by the NOESY data, which showed the following key correlations: H-6/H-9, H-2/CH_3_-14 and H-5/CH_3_-14 (Figure 2). Compound **5** represents a pyrroloquinoline alkaloid that was obtained naturally for the first time. Although the structure of **5** was previously synthesized to determine the constitution of calycanthine using the alkaloid degradation method, no spectroscopic data were previously reported.^20^ The regioisomeric alkaloid of compound **5**, known as marinoquinoline A, was previously isolated from the gliding bacterium *Ohtaekwangia kribbensis*.^21^ The structures of **5** and marinoquinoline A are identical except for the location of the methyl group: in compound **5**, the methyl group is attached to the pyrrole nitrogen atom, while in marinoquinoline A, it is attached to C-2. Additionally, the ^1^H and ^13^C NMR data for both compounds are largely consistent with the most significant deviations in chemical shifts observed at C-2 and N-Me/NH.

**Figure 2:**
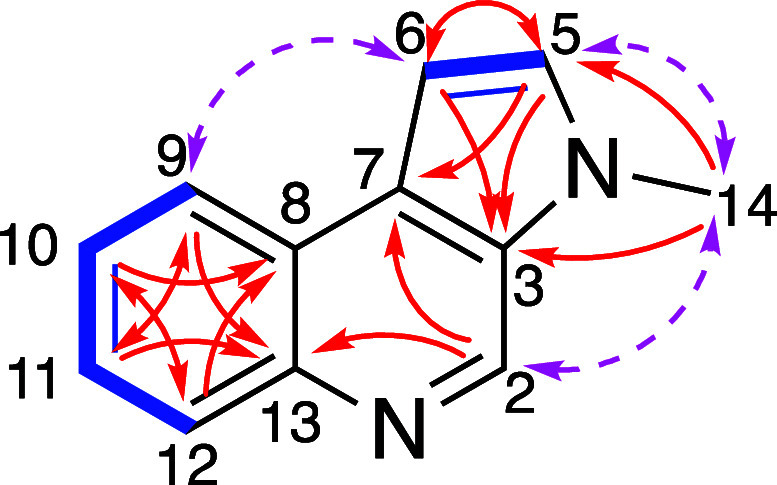
Selected COSY (blue bold bonds), HMBC (red arrows), and NOESY (pink dotted arrows) correlations of **5**.

**Table 1:** ^1^H and ^13^C NMR Spectroscopic Data of Eumatricine (5) in CDCl_3_ ^a^

position	δ_H_ (mult, *J* in Hz)	δ_C_ (ppm)
**2**	9.04, s, 1H	136.1
**3**		128.9
**5**	7.27, d (2.9), 1H	130.8
**6**	7.00, d (2.9), 1H	100.2
**7**		129.8
**8**		123.5
**9**	8.20, dd (7, 2), 1H	123.0
**10**	7.58, td (7, 2), 1H	125.9
**11**	7.60, td (7, 2), 1H	126.1
**12**	8.18, dd (8, 2), 1H	129.6
**13**		142.4
**14**	4.04, s, 3H	33.5

a ^1^H NMR and ^13^C NMR were measured at 600 and 150 MHz.

Since the genus *Palicourea*, a relative of *Eumachia*, is also a producer of β-carboline alkaloids, eumatricine (**5**) is therefore assumed to be derived from a β-carboline precursor.^22,23^ It is also known that a β-carboline alkaloid (pyridoindole, 6–5–6 ring system) can undergo a ring expansion rearrangement to a pyrroloquinoline alkaloid (6–6–5 ring system).^24^ A plausible biosynthetic pathway to **5** starting from the β-carboline alkaloid tryptoline was proposed as demonstrated in Figure 3. First, oxidative cleavage of the C-2–C-7 double bond gives the dicarbonyl intermediate i, which, following an amide reduction-dehydration step, gives the enamine intermediate ii. Nucleophilic enamine addition onto the ketone at C-7 then gives the 6–6–5 tricyclic intermediate iii. The imine function at C-2 is then converted to an amide group, while the hydroxyl group at C-7 is dehydrated to form a tetrasubstituted double bond at C-3 and C-7, giving rise to the intermediate iv. Finally, oxidative dehydrogenation at C-5 and C-6 followed by methylation of the pyrrole N atom furnishes the structure of eumatricine (**5**).

**Figure 3:**
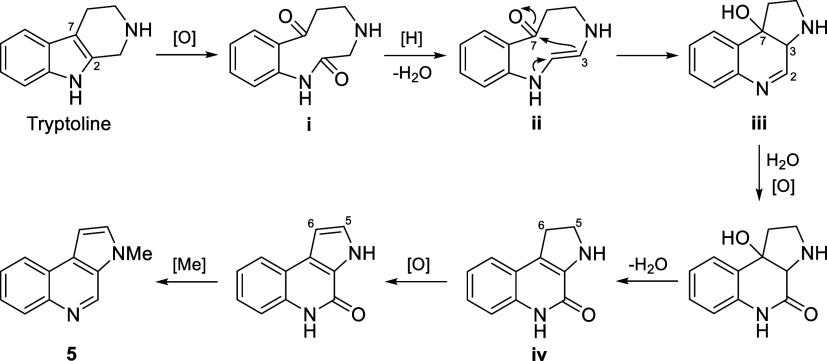
Plausible biosynthetic pathway to eumatricine (**5**).

**Figure 4:**
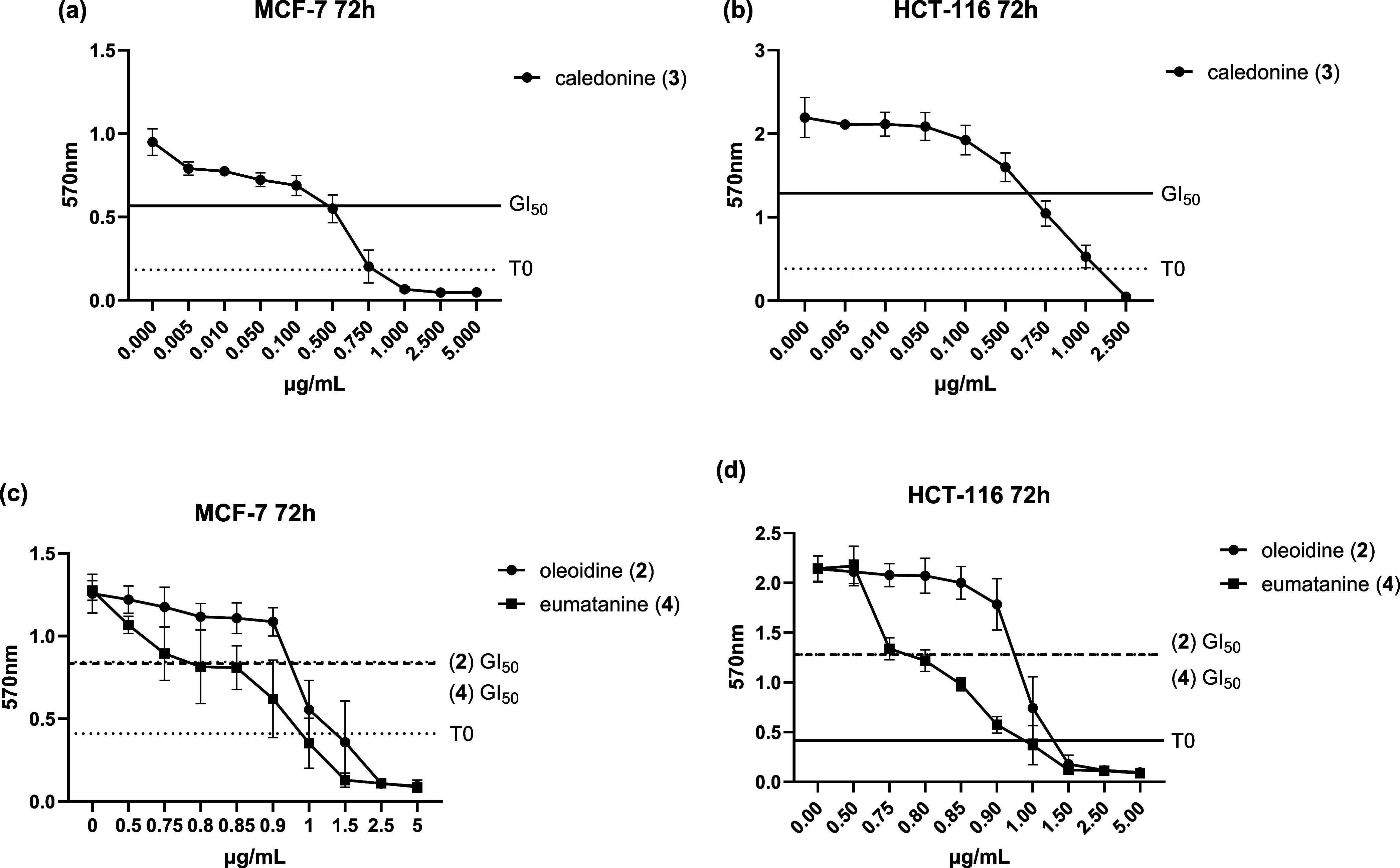
Growth inhibitory effects of caledonine (**3**) (a and b),as well as oleoidine (**2**) and eumatanine (**4**) (c and d) on MCF-7, HCT-116 cell lines. Cells were seeded on 96-well plates and incubated for 72 h (*n* = 6 internal repeats, ≥3 independent trials). The line of T0 represents the number of cells treated, while GI_50_ represents the concentration of test agents that inhibit the growth of cells by 50%.

### Preliminary Screening

MTT assays showed that oleoidine (**2**), caledonine (**3**), and eumatanine (**4**) elicit potent broad-spectrum growth inhibition against 8 human-derived cell lines with GI_50_ values ranging from 0.1 to 2.7 μM. Table 2 presents the mean GI_50_ values of **2**, **3**, and **4** against the 8 human-derived cell lines, including carcinoma cell lines MCF-7, MDA-MB-468 (breast), HCT-116, HT-29 (colon), U373 (GBM, vector control), U373 M (GBM, MGMT-transfected), A549 (nonsmall cell lung cancer) and nontransformed lung fibroblasts MRC-5 (Table 2); activity was corroborated by cell count assays and clonogenic cytotoxicity (ability to survive brief challenge and form progeny colonies) assays (Figure 5b–d and Figure 6).

**Table 2:** Effects of Compounds **2**, **3**, **4** on the Growth of Cells^a^

		compounds GI_50_ mean ± SEM (μM)		
origin	cell lines	oleoidine (2)	caledonine (3)	eumatanine (4)
breast carcinoma	MCF-7	1.204 ± 0.148	0.300 ± 0.044	0.706 ± 0.105
	MDA-MB-468		0.257 ± 0.023	
colon carcinoma	HCT-116	0.950 ± 0.022	0.585 ± 0.032	0.618 ± 0.102
	HT-29		2.707 ± 0.508	
brain carcinoma	U373 V		0.137 ± 0.045	
	U373M		0.117 ± 0.040	
lung carcinoma	A549		0.622	0.531 ± 0.081
nontransformed lung fibroblast	MRC-5	0.943 ± 0.006	0.877 ± 0.191	0.630 ± 0.058

a MTT assays, following 72 h exposure of cells to compounds **2**, **3**, **4**; mean ± SEM GI_50_ values (μM) are values from ≥ 3 independent trials where *n* = 6 internal repeats, except that of A549, only 1 independent trail was performed on compound 3 and 2 on compound **4**, where *n* = 6.

**Figure 5:**
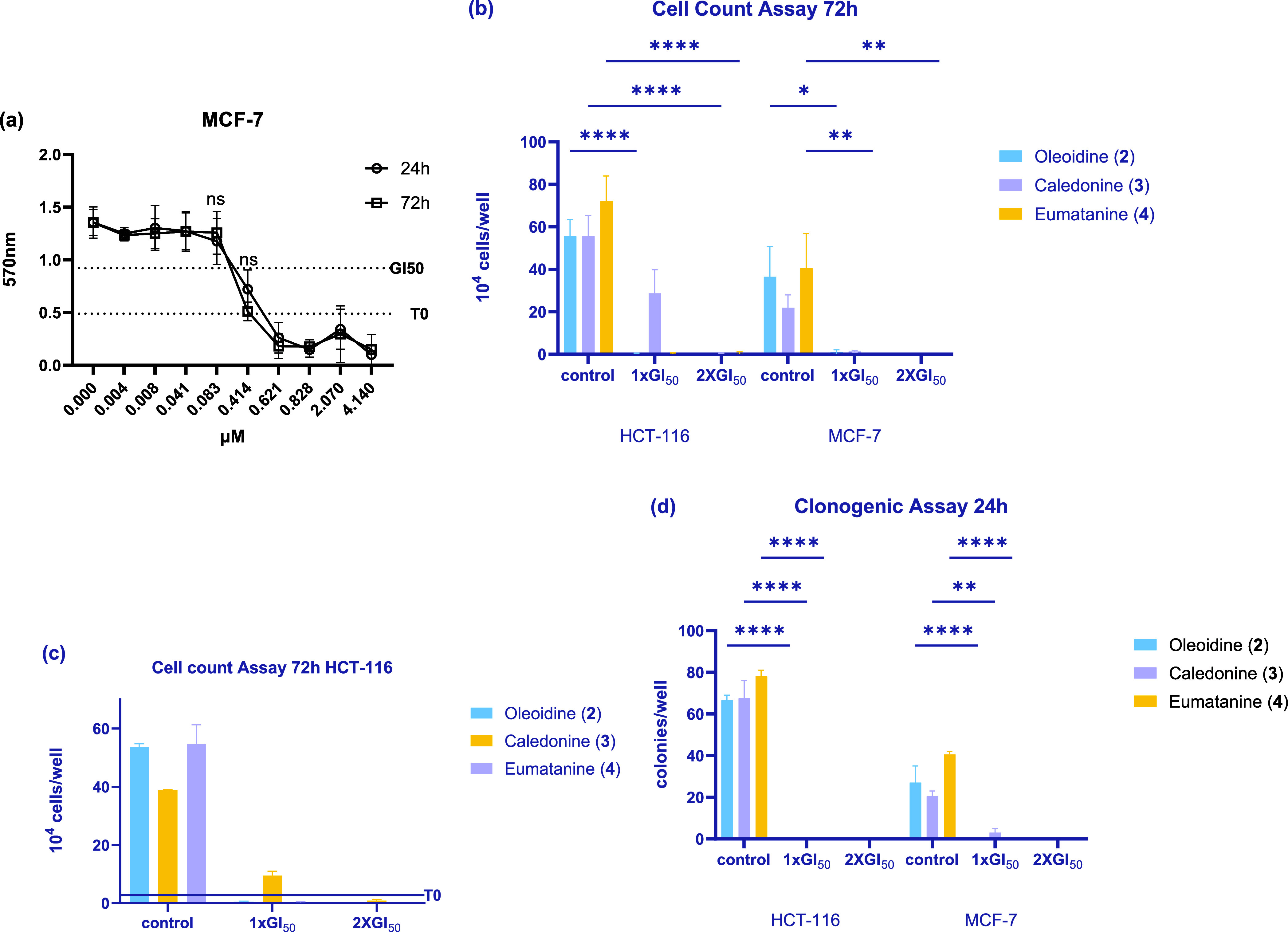
(a) Growth inhibitory effect of caledonine (**3**) on MCF-7 (24 and 72 h); mean ± SEM from ≥3 independent trials where *n* = 6. ns, *p* > 0.05 24 h vs 72 h. (b) Effect of compounds **2**, **3**, and **4** on cell growth (72 h); mean ± SEM from ≥3 independent trials where *n* = 2. (c) Representative results of growth inhibitory effects of compounds **2**, **3**, and **4** on HCT-116. (d) Effect of compounds **2**, **3** and **4** on colony formation (24 h); mean ± SEM from ≥3 independent trials where *n* = 2. (*p < 0.05, **p < 0.01, ***p < 0.001, ****p < 0.0001 vs the control group).

**Figure 6:**
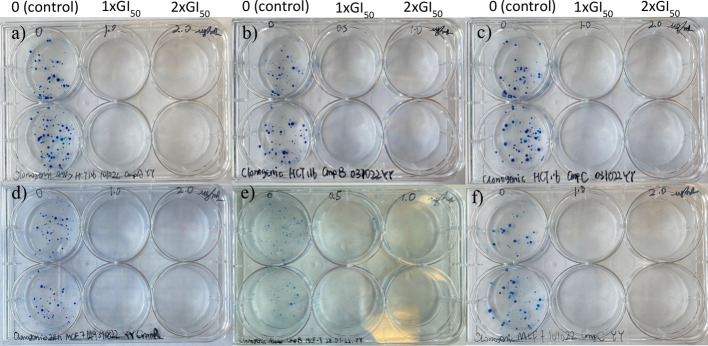
Representative photograph showing the effect of compounds **2**, **3**, and **4** on HCT-116 (a–c) and MCF-7 (d–f) colony formation at 0, 1 × GI_50_ and 2 × GI_50_.

It is indicated that the GBM cell lines are most -and ∼equi-sensitive to compound **3** among all cancer cell lines - GI_50_ **3** < 0.14 μM against U373 V and U373M, which implies that the activity of **3** is not influenced by *O*6-methylguanine-DNA methyltransferase (MGMT) gene expression (Figure 4). The DNA repair enzyme MGMT is capable of reversing *O*6 methyl guanine (*O*6MG) damage caused by alkylating agents (such as Temozolomide, the standard of care agent for GBM treatment in the clinic) via a one-step transalkylation reaction, which is one of the major chemoresistance mechanisms of Temozolomide.^25,26^ However, the ability of MGMT to reverse *O*6MG damage relies on continuous MGMT expression, and multiple methods are available for the regulation of MGMT, including promoter methylation, altered expression, histone modifications, post-transcriptional modifications, and miRNA regulation of transcription levels.^27,28^ It is likely that **3** causes cytotoxicity via pathways other than inducing the lesion of *O*6MG; potentially, however, **3** may be a MGMT regulator. For example, the inhibition of the wnt/β-catenin pathway decreases MGMT gene expression, thereby preventing chemoresistance in response to Temozolomide as well as increasing drug sensitivity.^29^ This may be especially advantageous under specific clinical conditions, as the overexpression of MGMT in glioma is currently a major obstacle to successful treatment, portending poor prognoses.

Although compounds **2**, **3**, and **4** have shown potent activity toward various cancer cell lines, the GI_50_ values against nontransformed lung fibroblasts MRC-5 cells may suggest that compounds **2** and **4** are not cancer-selective; only **3** indicated slight cancer-selectivity in GBM (selectivity index (SI) > 6) and breast cancer (SI > 2.5) cell lines. Possible techniques to circumvent the potential cytotoxicity of anticancer agents toward normal cells include nanoparticle formulations and antibody-drug conjugates (ACDs). For instance, pegylated liposomal doxorubicin (Doxil) was the first FDA-approved nanodrug (1995), encapsulating the anticancer agent doxorubicin in liposomes for drug delivery.^30^ Doxorubicin, a natural product (anthracycline antibiotic) originally isolated from microorganisms (*Streptomyces peucetius*), possesses potent broad-spectrum anticancer activity and has been used clinically for decades, but is plagued by cardiotoxicity as its major side effect.^31–33^ Doxil has displayed multiple advantages over conventional doxorubicin, including prolonged drug circulation time, reticuloendothelial system (RES) evasion, and enhanced cancer-selectivity.^30^ In addition, enhanced permeation and retention (EPR) may also allow a lower dose administration and thus minimize systemic-toxicity, reducing adverse side effects, specifically, doxorubicin-induced cardiotoxicity, which is dose-dependent, cumulative, and irreversible.^32^

ADCs are another new class of therapeutic formulations that enable cytotoxic agents (e.g., NPs) to be harnessed therapeutically, the targeting abilities by combining with cancer-specific monoclonal antibodies (mAbs).^34^ To date, 13 ADCs (such as Mylotarg, Adcetris, Kadcyla, and Trodelvy) have been approved by the FDA to treat cancer, suggesting tremendous promise for ADCs in targeted cancer therapies. Kadcyla, a clinically used ADC for HER2+ breast cancers, combines the humanized monoclonal antibody trastuzumab (Herceptin) with the cytotoxic agent DM1, a derivative of the natural product maytansine.^35^ Trastuzumab selectively delivers DM1 to tumor cells by binding to the HER2 receptor while retaining its ability to inhibit HER2 signaling and activate antibody-dependent cell-mediated cytotoxicity.^35,36^ Maytansine, originally isolated from *Maytenus serrata*, is a potent antimicrotubule agent, with DM1 being 25- to 270-fold more cytotoxic than paclitaxel and 180- to 4,000-fold more cytotoxic than doxorubicin.^36–38^ Apoferritin (AFt) represents a promising ADC delivery vehicle with distinct advantages in overcoming mechanisms of drug resistance mediated by ATP-binding cassette protein pumps, such as P-glycoprotein (Pgp).^39^ These pumps, which have coevolved to mitigate damage from toxic natural products, are key contributors to multidrug resistance (MDR). For instance, AFt-encapsulated jerantinine A demonstrated significantly enhanced anticancer activity and improved cancer selectivity in a panel of breast cancer cell lines.^39,40^

The comparison between 24 and 72 h exposure of cells (MCF-7; HCT-116) to **3** inferred rapid onset cytotoxicity (Figure 5a). Differing exposure periods during the same overall experimental time (i.e., 24 h treatment followed by 48 h test agent-free vs 72 h continuous exposure of cells to test agent; Figure 5a), revealed that **3** exerts almost identical growth inhibitory activity after 24 or 72 h treatment; the slightly higher potency at 72 h treatment at concentrations ∼GI_50_ value is not significant (ns, *p* > 0.05 24 h vs 72 h). This confirms the rapid onset of cytotoxicity caused by **3**. In cell count and clonogenic assays (Figure 5b–d), exposure to all three compounds at 2 × GI_50_ resulted in a significant reduction in the number of viable cells, further corroborating their potent cytotoxic nature.

### Assessment of Cellular Mechanisms

After confirmation that compounds **3** and **4** possess the ability to inhibit cancer cell proliferation and colony formation, their ability was further evaluated in MCF-7 and HCT-116 cells to detect their cellular mechanisms. Cell cycle analyses revealed that **4** increased pre-G1 events in both cell lines dose- and time-dependently, in which a significant increase (*p* < 0.0001) was recorded in both cell lines at concentrations of ∼2 × GI_50_ after 72 h treatment and in HCT-116 for 24 h treatment (Figure 7). Notably, **4** even induced a significant increase (*p* < 0.0001) in pre-G1 events at a concentration of ∼1 × GI_50_ after 72 h exposure in MCF-7 cells (Figure 7d). However, there was no cell phase-specific cell cycle arrest observed in either HCT-116 cells under all conditions or MCF-7 cells when the treatment time was 72 h. Instead, significant G1 phase arrest was observed (*p* < 0.05) in MCF-7 cells after 24 h exposure at 1 × GI_50_, meanwhile just a slight increase was observed in the pre-G1 events (ns, *p* = 0.9825) (Figure 7c), which may be due to greater initial seeding density for 24 h exposure (2 × 10^5^ for 24 h assays, 1 × 10^5^ for 72 h assays), and thereby compounds added to give the same final concentration may exert a lower level of cytotoxicity on cells. Given that the same experimental conditions were applied, compound **4** likely exerts cytotoxic effects in HCT-116 and MCF-7 cells through distinct cell death mechanisms, potentially due to MCF-7 cells being caspase-3 null, which may prevent rapid apoptosis and instead trigger alternative pathways such as paraptosis or autophagy in response to oxidative stress and DNA damage.^41^

**Figure 7:**
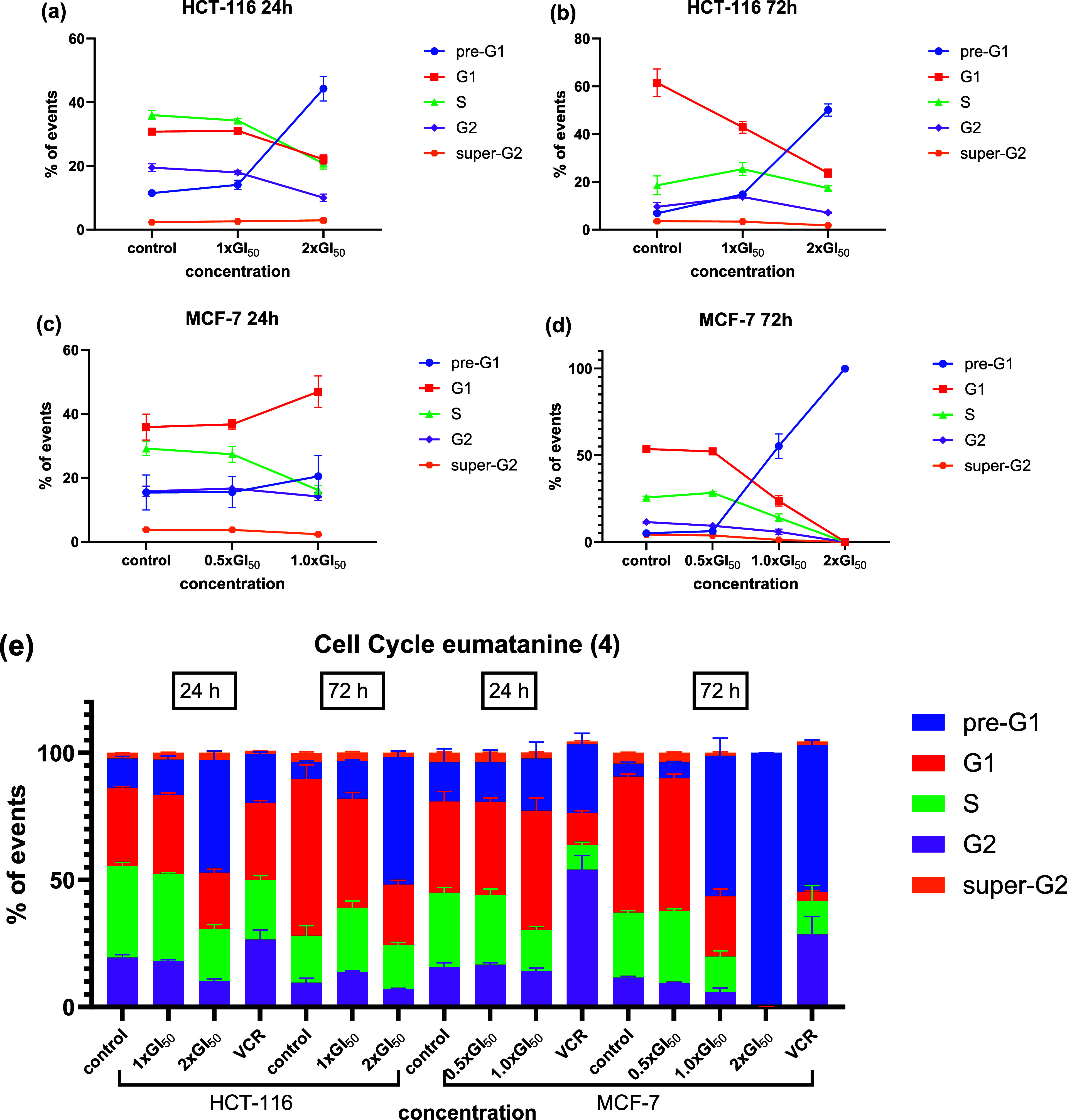
Summary of the effect of 1 × and 2 × six-well-plates (control dishes were treated with blank media); mean ± SEM from 3 independent trials where *n* = 2; 10,000–50,000 cells/events were analyzed per GI_50_ eumatanine (**4**) (24 and 72 h) on HCT-116 and 0.5 ×, 1 ×, and 2 × GI_50_ **4** (24 and 72 h) on MCF-7 cell cycle; cells were seeded at the density of (1–2) × 10^5^ cells/well using sample.

Vincristine, an anticancer alkaloid derived from *Catharanthus roseus* (L.) G. Don (Madagascar periwinkle) was adopted as a positive control, causing significant G2 phase arrest in accordance with its microtubule-disruptive mechanism of action; Figure 7.^42^ Notably, pre-G1 phase accumulation (<2N-DNA) and sustained G1-phase arrest were also observed in HCT-116 and MCF-7 cells by the addition of conofolidine, a bisindole alkaloid from the Malayan plant *Tabernaemontana corymbosa*, along with caspase activation, ROS generation, and DNA damage. Interestingly, induction of apoptosis or senescence was observed in multiple carcinoma cell lines (including breast, colon, and lung cancer).^43^ This demonstrates that NPs may induce distinct cell fates; similarly, eumatanine (**4**) may trigger multiple cellular mechanisms, resulting in cell death.

G1/S and G2/M are two important checkpoints in the cell cycle that police DNA integrity, ensuring that damaged DNA is either repaired or cells with irreparable DNA damage are instructed to undergo programmed cell death (e.g., apoptosis). Upon detection of DNA damage, cell cycle arrest ensues to stabilize the genome and prevent uncontrolled proliferation.^44,45^ Additionally, the G1/S checkpoint also ensures that the cell has adequate nutrients and growth factors for subsequent cell growth and division.^44^ The retinoblastoma (RB) protein (e.g., pRb, p107 (RBL1), and p130 (RBL2)) and transcription factor p53 are known as central tumor suppressors; both play an important role in the induction of apoptosis, senescence, and the regulation of cell cycle, particularly in the G1 phase.^46,47^ They crosstalk with cyclin-dependent kinase inhibitor p21/CDKN1A and manipulate transcription of genes that are involved in a variety of cell functions (e.g., MAP kinase MAP3K5/ASK-1 for cell division, the topoisomerase-binding protein TOPBP1 for DNA replication and repair) via p53-p21-RB signaling.^47^

RB modulates DNA replication and G1/S transition during the cell cycle by negatively regulating two positive regulators of cell cycle entry, namely cyclin-dependent kinases (CDKs) and transcription factors E2F, while the interaction with E2F to form RB-E2F complexes is essential for repressing G1/S cell cycle genes (e.g., CDK1), DNA polymerase α (POLA1), cyclin A (CCNA2), thymidine kinase (TK1), dihydrofolate reductase (DHFR), and minichromosome maintenance complex component 3 and 5 (MCM3/5, DNA replication licensing factors) that are responsible for DNA synthesis, DNA replication and DNA repair.^46,48^ It is notable that loss of RB function is observed in many tumors by various mechanisms (e.g., RB1 mutation, RB proteolysis, loss of RB-E2F interaction), which gives rise to induction of cell division, defects in cell cycle exit, impaired ability to eliminate DNA damage and enter senescence, as well as positive regulation of cell-cycle-checkpoint control, particular at the G1/S transition, ultimately leading to cell cycle dysregulation and malignant proliferation.^47,49^

P53, a widely studied tumor suppressor capable of inducing cell cycle arrest, and contributing to DNA repair, metabolic pathways’ regulation, senescence, and apoptosis induction, with the underlying mechanisms of transcriptional regulation of cell cycle genes (e.g., the apoptosis inducers BAX, PUMA/BBC3).^50,51^ Similar to RB, p53 regulates the G1/S transition by repressing genes (e.g., p21), and p53 function is lost in >50% of tumors, either consequence of p53 mutations, or disruption of the p53 pathway, for example the proteolysis of p53 induced by viral oncoproteins or MDM2 overexpression.^47,52^ In the MTT assay, compound **3** exhibited potent cytotoxicity across all cell lines, with GI_50_ values being slightly higher in wild-type p53-expressing cell lines (HCT-116, MCF-7, A549; range from 0.4–0.6 μM) compared to mutant p53-expressing cell lines (MDA-MB-468, U373; range from 0.1–0.3 μM), suggesting that the activity of **3** is independent of p53 status.^43,53–55^

A pre-G1 population usually represents cells undergoing apoptotic cell death, and therefore, further apoptosis assays are necessary to confirm the induction of apoptosis by compound **4**. Significant late-stage apoptotic cell death was recorded in MCF-7 and HCT-116 cells by annexin V-FITC/PI (propidium iodide) apoptosis assays after 24 h (2 × GI_50_, ∼1.449 μM, *p* ≤ 0.0002) or 72 h (1 × GI_50_, ∼0.725 μM, *p* ≤ 0.0001) exposure to **4**, verifying the apoptotic cell death recorded in cell cycle analysis (Figure 8a,b). Following 72 h exposure, 2 × GI_50_ **4** induced higher levels of apoptosis in both cell lines when compared to 5 × GI_50_ vincristine (Figure 8b). Enhanced apoptosis in breast and colon cancer cells may indicate that the apoptotic signaling pathways have been re-established, and NP **4** has the potential to eradicate cancer cells. Possible reasons for not detecting cell cycle arrest should be considered, such as the compound may not be cell cycle specific, the extent of DNA damage being catastrophic enough to trigger immediate cell death, or cell death may not only be caused by apoptosis but also a consequence of nonapoptotic cell death modes (e.g., senescence, paraptosis or autophagic cell death).

**Figure 8:**
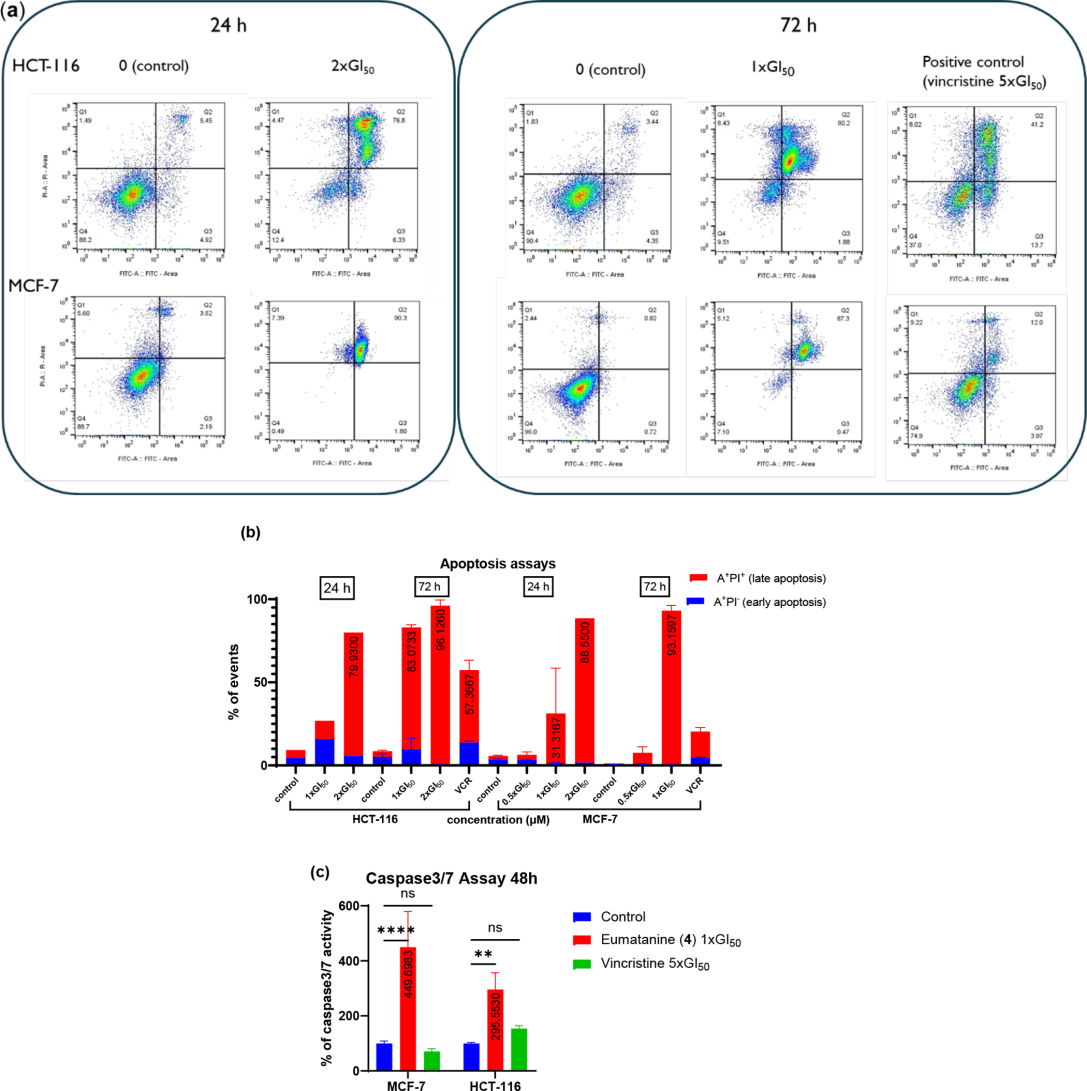
(a) Effect of ∼0.363, ∼0.725, and ∼1.449 μM eumatanine (**4**) (24 and 72 h) on HCT-116 and MCF-7 apoptosis; *X*-axis is FITC, *Y*-axis is PI; ≥3 independent trials performed where *n* = 2, except only one trial was performed for HCT-116 vs 4–24 h and MCF-7 vs 4–24 h at 2 × GI_50_, and two trials were performed for MCF-7 vs 4–24 h at 0.5 × GI_50_ ; >10,000 events recorded per sample; (b) summary of the effect of ∼0.363, ∼0.725, and ∼1.449 μM eumatanine (4) (24 and 72 h) on HCT-116 and MCF-7 apoptosis; (c) Effect of ∼0.725 eumatanine (4) (48 h) on HCT-116 caspase activity; mean ± SEM from ≥3 independent trials where *n* = 3. (ns *p* > 0.05, *p < 0.05, **p < 0.01, ***p < 0.001, ****p < 0.0001 vs the control group).

NP honokoil, a biphenolic compound isolated from *Magnolia*, exerts anticancer activity by inducing apoptosis and autophagy in various cancer cell lines (e.g., ovarian cancer cells, human osteosarcoma cells, pancreatic cancer cells, and B-cell chronic lymphocytic leukemia (B-CLL) cells) without causing cell cycle arrest in G1, S, or G2/M phases.^56–59^ Instead, honokiol causes an accumulation of events in the pre-G1 cell cycle phase and induces apoptosis via a variety of mechanisms, including modulation of adenosine monophosphate-activated protein kinase (AMPK), the ROS/ERK1/2 and the NF-κB signaling pathways, along with activation of caspase-3, -7, and -9, PARP cleavage, as well as the generation of ROS.^56–59^

Members of the caspase family of aspartic-acid-specific proteases play a key role in the execution of apoptotic cell death. Caspases include the upstream initiators caspase-8, caspase-9, and caspase-10 in humans, as well as the downstream effectors caspase-3, caspase-6, and caspase-7.^60^ It has been reported that >400 substrates are proteolyzed by apoptosis-associated caspases, among which are caspase-3 and caspase-7 major executioner caspases.^61^ The result of the caspases-3/7 assay illustrated that the activities of caspases-3/7 were significantly (*p* < 0.01) induced by **4** at 1 × GI_50_ (∼0.725 μM) in MCF-7 and HCT-116 cells after 48 h exposure, further confirming that **4** caused apoptotic cell death in both MCF-7 and HCT-116 cells (Figure 8c). Many anticancer drugs that are derived from NPs induce apoptotic cell death by activating caspases, including doxorubicin and cisplatin, which are known to induce apoptosis via ROS generation and subsequent caspase activation.^33,62^ Interestingly, although it is known that MCF-7 cells lack caspase-3, a significant increase in the activity of casapase-3/7 is observed in MCF-7; similarly, the styrylpyrone derivative (SPD) induced apoptotic cell death in MCF-7 cells through a caspase-7-dependent pathway.^41,63^

Evasion of apoptosis or apoptosis-resistance is a hallmark of cancer.^64^ Imbalance between pro-apoptotic signals (e.g., cytochrome-*c*, AIF, and death receptors TNFR1, Fas) and antiapoptotic signals (e.g., Bcl-2 (group I), c-FLIP, IAP) is a vital mechanism for malignant cells to evade apoptosis.^65^ Other mechanisms include decreased caspases̀ activity and blocked death receptor signaling.^66,67^ Apoptosis-evasion contributes to carcinogenesis, and apoptosis-induction is a valid strategy to treat cancer. Therapies capable of re-establishing apoptotic signaling pathways, enhancing apoptosis, or inducing alternative mechanisms of cancer cell death hold significant potential for effective cancer therapy.

Principally, apoptosis follows either intrinsic pathways (mitochondrial pathway and endoplasmic reticulum (ER) pathway) or extrinsic pathways (death receptor pathway), triggered by cellular stress, DNA damage, hypoxia, and immune surveillance mechanisms.^68^ The intrinsic mitochondrial pathway, as the most common mechanism of apoptosis in vertebrates, can be positively regulated by proapoptotic BCL-2 family members such as BAX, BAK, BIM, BID, PUMA, and negatively regulated by the antiapoptotic BCL-2 family members such as BCL-XL, Bcl-2, A1, and MCL1.^69^ On the other hand, BCL-2 family proteins modulate mitochondrial outer membrane permeabilization (MOMP), and normally, BAX and BAK trigger MOMP with the release of cytochrome *c* into the cytoplasm, which can engage caspase-9 and activate caspases-3 and 7, ultimately leading to apoptosis.^69–71^ Simultaneously with the release of cytochrome *c*, two other proapoptotic proteins are released from mitochondria, namely Smac and Omi, which can engage caspase inhibitor X-linked inhibitor of apoptosis (XIAP; Figure 9).^72^

**Figure 9:**
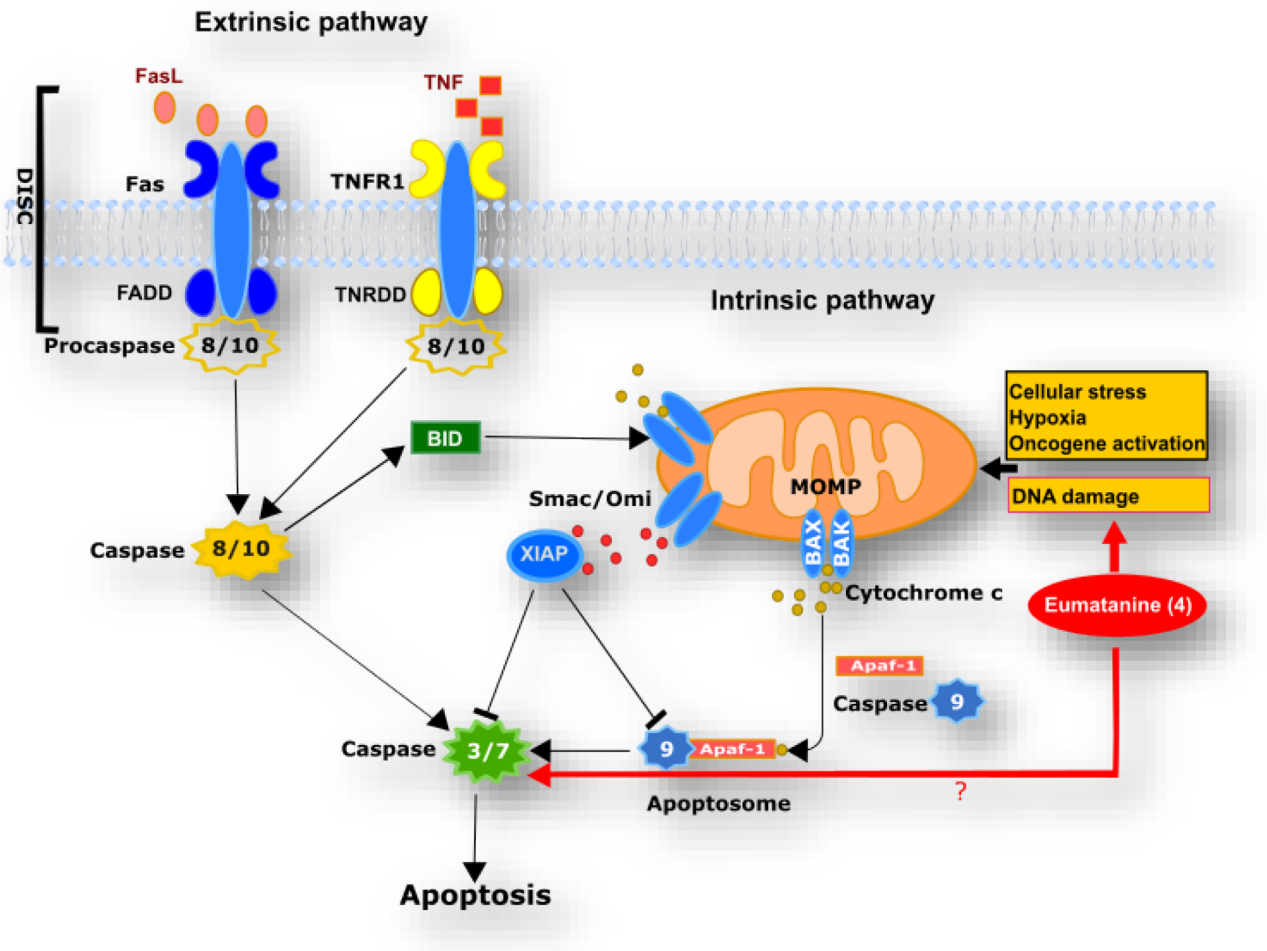
Extrinsic and intrinsic apoptotic pathways. The extrinsic pathway is activated through the binding of death receptors, such as Fas and TNFR1, to their corresponding ligands, including FasL and TNF. This interaction recruits the adaptor protein FAS-associated death domain (FADD) and the initiator procaspases-8 and −10, culminating in the formation of the death-inducing signaling complex (DISC). The assembly of DISC facilitates the dimerization and subsequent autoactivation of the initiator caspases. These activated caspases then cleave and activate the executioner caspases-3 and -7, driving the apoptotic process unless inhibited by X-linked inhibitor of apoptosis protein (XIAP). The intrinsic pathway is initiated in response to various intracellular stress signals that influence interactions among BCL-2 family proteins, ultimately regulating the activation of the BCL-2 effector proteins BAX and BAK. Once activated, BAX and BAK induce mitochondrial outer membrane permeabilization (MOMP), resulting in the release of proapoptotic proteins from the intermembrane space (IMS). Among these, cytochrome *c* (Cyt c) binds to APAF1, promoting its oligomerization and leading to the formation of the apoptosome. This complex recruits and activates initiator caspase-9, which subsequently cleaves and activates the executioner caspases-3 and -7. Concurrently, Smac/DIABLO is released from the mitochondrial intermembrane space (IMS) along with Cyt *c* and acts to inhibit the X-linked inhibitor of apoptosis protein (XIAP), thereby promoting apoptosis. The intrinsic and extrinsic pathways are interconnected, as caspase-8 from the extrinsic pathway can cleave the BH3-only protein BID, producing its active form, which subsequently activates BAX and BAK, leading to intrinsic apoptosis. Eumatanine (**4**) has been shown to activate caspases-3/7 and induce DNA damage, as reported in this study. However, the specific cell death pathway involved remains unclear.

The extrinsic pathway initiates apoptosis by the ligation of death receptors (including proapoptotic death receptors TNFR1, Fas and TRAIL-DR4 and -DR5) and corresponding ligands at the cell membrane, followed by the recruitment of adaptor proteins such as Fas-associated death domain protein (FADD) and the activation of apoptosis-initiating protease caspases-8 and -9 (Figure 9).^73–75^ In this pathway, caspase-8 or 10 can then either cause the cleavage of the BH3-only protein BID and thereby drive the mitochondrial intrinsic pathway via BAX and BAK (mainly), or engage apoptosis by activating the executioner caspases-3 and -7 (minorly) (Figure 9).^68,76^ The p53 tumor suppressor can induce cell death (including cell cycle arrest, apoptosis and senescence) via both the extrinsic and intrinsic cell death pathways by regulating death receptors (such as Fas, DR5) in response to DNA damage, hypoxia, viral infection, or oncogene activation, and therefore the stabilized p53 protein is an important target of cancer therapy.^77^ Additionally, the extrinsic pathway can be triggered by immune mechanisms (e.g., the expression of TRAIL (induced by interferon action) and cell death ligands (e.g., PD-L1) on natural killer (NK) cells and thereby lead to cell death.^78,79^ Various other cellular processes, such as autophagy, anoikis, ferroptosis, and regulated necrosis, can crosstalk with apoptotic signaling pathways in response to cancer therapeutics (e.g., proapoptotic agents).^68^

Reactive oxygen species (ROS), such as superoxide anion (O_2_^–^), hydrogen peroxide (H_2_O_2_), singlet oxygen, hydroxyl radical, and peroxy radical, arise from intracellular metabolic processes or external insult, mediating cell proliferation, differentiation, and migration, while playing a beneficial role in regulating normal cellular processes (i.e., redox signaling) and being implicated in physiological processes such as glucose homeostasis, inflammation, cellular lifespan, and the progression of diseases like carcinogenesis, cardiovascular disease, and neurodegeneration.^80–83^ The role of excess ROS, such as H_2_O_2_, has been connected to genomic (in)­stability, regulation of transcription, and signal transduction, which may contribute to both tumor progression and cancer cell death.^84^ H_2_O_2_, as the most stable ROS generated in cultured cells, has been utilized as a sensitive biomarker to detect the ROS levels within cells and thus to evaluate how intracellular oxidative stress is affected by tested compounds or certain specific conditions.^85^

Production of ROS was measured in HCT-116 and MCF-7 cell lines following exposure to either **4** or vincristine. Significantly (*p* < 0.01) enhanced ROS were generated by **4** in treated HCT-116 (140%) and MCF-7 (174%) cells after 24 h exposure at 1 × GI_50_ (∼0.725 μM) (Figure 10a). ROS are regarded as an important biomarker in cancer development that contribute not only to oncogenesis by the mediation of oxidative DNA damage, but also serve as targets for promising therapeutic strategies, such as modulating cellular redox status to manage cancer when ROS levels become excessive.^86^ A significant number of natural anticancer agents generate ROS, an obligate facet of their mechanisms of action, and induce apoptosis in cancer cells, including etoposide, doxorubicin, taxanes (microtubule-stabilizing agents), cisplatin (an alkylating agent), and jerantinine B, with etoposide and doxorubicin being FDA-approved topoisomerase II inhibitors that cause DNA damage and evoke G2/M cell cycle arrest.^33,40,87–93^ Interestingly, doxorubicin caused both G1/S and G2/M arrest in MCF-7, NP **4** induced G1 phase arrest only after short-term exposure (24 h) in MCF-7 cells, but caused no cell cycle arrest in HCT-116 cells, suggesting that doxorubicin and **4** may trigger similar mechanisms in MCF-7 cells, and also suggesting that NP **4** may trigger different mechanisms in distinct cell lines—according to their molecular machinery.^94^ Bar-on et al. reported that doxorubicin behaves differently in different breast cancer cell lines, causing both G1/S and G2/M phase arrest in MCF-7, while inducing only G2/M phase arrest in MDA-MB-231. Additionally, in MCF-7, Skp2 levels significantly decreased, p27 levels slightly reduced, and p53 and p21 levels increased, whereas in MDA-MB-231, p27 levels remained unchanged and cyclin B levels significantly increased, with the differential Skp2 expression attributed to arrest in different checkpoints of the cell cycle.^94^

**Figure 10:**
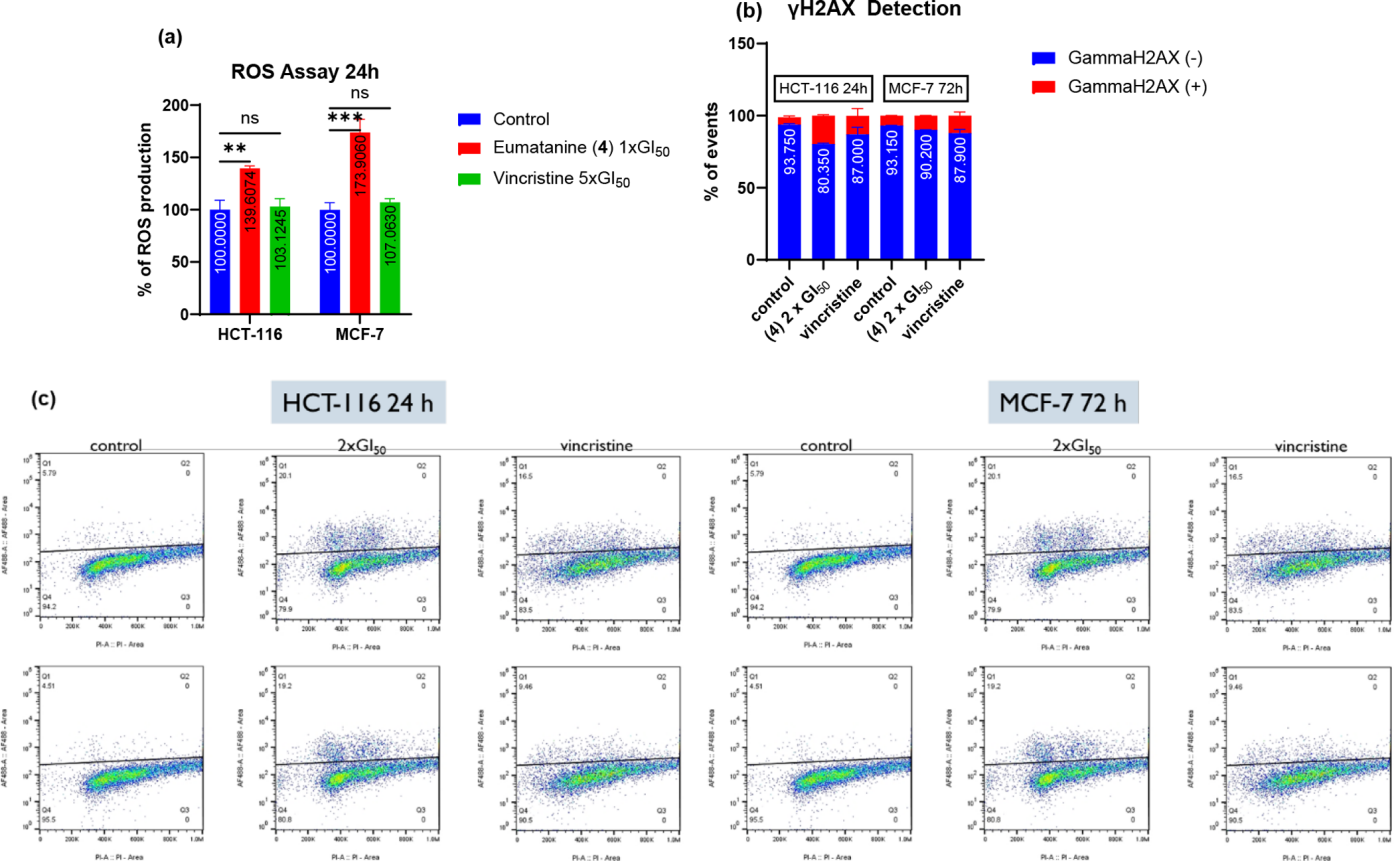
(a) Effect of 1 × GI_50_ eumatanine (**4**) (24 h) on HCT-116 and MCF-7 ROS; mean ± SEM from ≥3 independent trials where *n* = 2 (ns *p* > 0.05, *p < 0.05, **p < 0.01, ***p < 0.001, ****p < 0.0001 vs the control group.); (b) summary of γH2AX detection of 2 × GI_50_ (∼1.449 μM) eumatanine (**4**) on HCT-116 (24 h) and MCF-7 (72 h) cells, ≥3 independent trials performed where *n* = 2; (c) γH2AX detection of ∼1.449 μM eumatanine (**4**) on HCT-116 (24 h) and MCF-7 (72 h); *X*-axis is PI, *Y*-axis is γH2AX/AF488; ≥3 independent trials performed where *n* = 2, >10,000 events recorded per sample.

Given that ROS induction is typically associated with DNA damage, as seen with doxorubicin and etoposide, it is important to determine whether compound **4** causes DNA damage. Doxorubicin induces DNA damage either by inhibiting topoisomerase IIb or by intercalating with the strands, the latter typically occurring in regions rich in G-C base pairs.^95–97^ The formation of doxorubicin-DNA adducts has been shown to activate DNA damage responses and induce nontopoisomerase II cell death.^98^ Detection of DNA double-strand breaks (DSBs) is a valuable diagnostic tool for identifying precancerous and cancerous cells and assessing cancer progression and therapeutic efficacy in carcinoma cells.^93^ The DNA DSB is a serious DNA lesion that can be caused by exogenous or endogenous factors, such as cytotoxic agents, ROS, metabolic processes, deficient repair, telomere erosion, and programmed biological processes, ultimately leading to cell death (e.g., apoptosis) through disturbance of genomic stability.^99^ To detect and quantify DNA DSB in cells, a primary antibody against γH2AX and a fluorescent secondary antibody were used, incorporated with flow cytometry, which is a sensitive method to allow the active distinction of cells based on a cell’s particle density and fluorescence.^100^ H2AX, regarded as a tumor suppressor, is a member of the H2A histone family and a key component in DNA repair.^101^ Serine C-4 phosphorylation of H2AX (γH2AX) forms in response to DNA double-strand break (DSB) damage, γH2AX (phosphorylated H2AX) accumulates, surrounding the break sites, enabling the detection of individual DSBs by use of a γH2AX antibody.^99,100^ Figures 10b,c show that **4** at 2 × GI_50_ triggered DNA DSB in HCT-116 and MCF-7 cells following 24 or 72 h exposure, suggesting that NP **4** caused cell death by disrupting DNA integrity.

### Fluorescence Microscopy

Although cell cycle analysis, apoptosis assays, and caspase 3/7 assays suggest that compound **4** induces DNA fragmentation and caspase 3/7 activation—characteristics of apoptosis—the Nomenclature Committee on Cell Death (NCCD) emphasizes that these biochemical markers should not be solely used to diagnose apoptosis, as morphological changes are more reliable indicators.^102^ Programmed cell death, indeed, apoptosis is not always caspase-dependent and can occur without oligonucleosomal DNA fragmentation, for instance, staurosporine induces both caspase-dependent and caspase-independent apoptosis.^103^

To observe morphological changes in MCF-7 and HCT-116 cells caused by **4**, fluorescence microscopy was conducted. The cytoskeleton of cells was dyed with phalloidin (presented as green), and nuclei were dyed with DRAQ5 (far-red DNA stain, presented as violet). Morphological phenomena observed include membrane blebbing, nuclear condensation, cytoplasmic swelling, and cytoplasmic vacuolization (Figure 11). Membrane blebbing and nuclear condensation may suggest that cell death is associated with apoptosis, which corroborates the results obtained from the apoptosis assay and cell cycle analysis.^104–106^ Even though it is confirmed that **4** induced apoptotic cell death in MCF-7, the morphological features of apoptosis, such as membrane blebbing and cell shrinkage, are missing (Figure 11), which may be attributed to the fact that MCF-7 cells lack caspase-3 and the cell death ensues independently of caspase-3 (e.g., caspase-7-engagement).^41^

**Figure 11:**
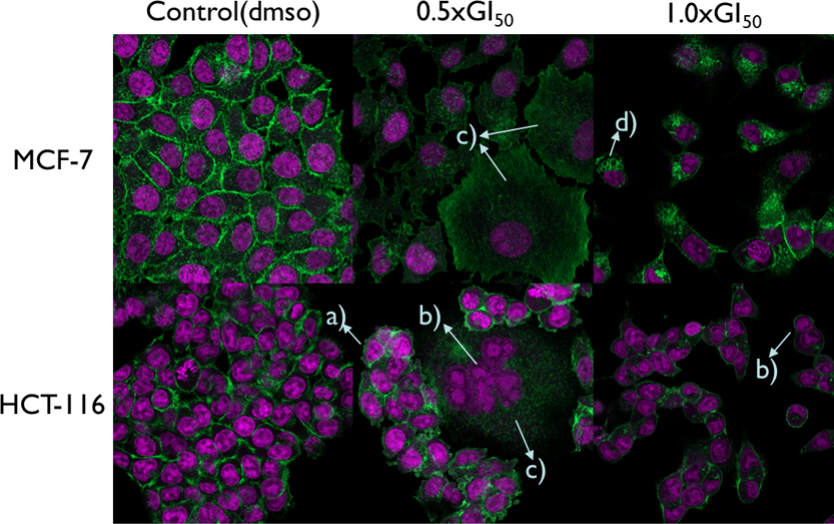
Effect of ∼0.363 and ∼0.725 μM eumatanine (**4**) (24 h exposure) on MCF-7 and HCT-116 cell morphology; (a) membrane blebbing; (b) nuclear condensation; (c) cytoplasmic swelling; (d) cytoplasmic vacuolization; ≥3 independent trials performed where *n* = 2; green dye represents cytoskeleton, violet dye represents nuclear contents.

Cytoplasmic vacuolization observed in 1.0xGI_50_ **4** treated MCF-7 cells may imply autophagy or paraptosis (Figure 11).^105,107^ Classical apoptosis typically begins with the collapse of cytoskeletal elements while organelles are preserved until the later stages, whereas autophagy starts with the degradation of organelles, with cytoskeletal elements remaining intact until the later stages.^107^ Autophagy, a regulated lysosomal degradation pathway, has been identified as the primary regulated mechanism for degrading long-lived proteins and the exclusive pathway for organelle degradation. Morphologically, autophagy is characterized by double-membraned vacuoles, which are formed to sequester cytoplasmic organelles such as mitochondria and ER.^107^ Paraptosis is a nonapoptotic form of programmed cell death mediated by mitogen-activated protein kinases (MAPKs) and does not activate caspases, with AIP-1/Alix identified as a specific inhibitor of this process.^105^ Cini et al. reported cytoplasmic vacuolization in MDA-MB-468 and HCT-116 cells following treatment with a titanium complex and attributed it to paraptosis, a caspase-independent programmed cell death route.^108^

Cytoplasmic swelling is an important morphological phenomenon of necrosis.^102,109^ Indeed, swelling in the cytoplasm of both MCF-7 and HCT-116 cells was observed after these cell lines were exposed to **4** at a concentration of 0.5 × GI_50_, indicating the induction of necrotic cell death occurred when exposed to a low concentration (∼0.363 μM) of **4** (Figure 11). Necrotic cell death, an alternative pathway when the classical caspase-dependent apoptotic pathway is inhibited and shares the same stimuli as apoptosis, is typically caused by mitochondrial oxidative phosphorylation, reactive oxygen species production, and noncaspase proteolytic cascades involving serine proteases, calpains, or cathepsins.^109,110^ Compared to the morphological features of apoptosis, such as cell shrinkage, extensive nuclear fragmentation, and intact plasma membrane until the later stages, necrosis is characterized by cytoplasmic swelling, rapid plasma membrane rupture, and organelle breakdown, with remarkably few changes in nuclear contents.^106,110^

Given that recent studies have shown autophagic cell death frequently occurs in diseased tissues and tumor cells in response to chemotherapy,^111^ it is understandable that combinations of morphological features of autophagic and apoptotic, or autophagic and necrotic, have been observed within the same cell; for instance, honokiol triggered both apoptosis and autophagy in human osteosarcoma cells.^56,107^ Morphological observations from **4** treated carcinoma cells in this study support engagement of multiple death pathways, demonstrating that features of autophagic/paraptotic cell death (cytoplasmic vacuolization) and necrotic cell death (cytoplasmic swelling) were observed in MCF-7 cells. On the other hand, morphological features of both apoptotic and necrotic cell death were observed in compound **4**-treated HCT-116 cells, further supporting the conclusion drawn from cell cycle analysis that different mechanisms of action are involved in MCF-7 and HCT-116 cells.

## Conclusions

In addition to the isolation of the known dimer (−)-calycanthine (**1**) and a novel tricyclic pyrroloquinoline alkaloid, eumatricine (**5**) from the leaves of *Eumachia montana*, a series of oligocyclotryptamine alkaloids were also isolated and characterized as the hexameric (+)-oleoidine (**2**), the heptameric (+)-caledonine (**3**), and the new octameric (+)-eumatanine (**4**), respectively. Preliminary assays suggested that **2**, **3**, and **4** exert potent broad-spectrum growth inhibitory and cytotoxic activity against carcinoma cell lines. Cell cycle analysis and apoptosis assays indicated that compound **4** cause rapid, cell cycle-phase-independent apoptotic cell death. Except for MCF-7 cells, significant G1 phase arrest is seen after a short exposure period (24 h). This may suggest that HCT-116 and MCF-7 engage different mechanisms of action in response to the 24 h treatment of **4**. Succinctly, **4** triggered apoptotic cell death through at least two pathways, including a caspase-3-dependent pathway in HCT-116 cells and a caspase-3-independent pathway in MCF-7 cells. Results obtained from the detection of ROS and γH2AX foci suggest that **4** causes cell death, at least in part, by generating ROS and evoking DNA DSBs. Finally, morphological changes, including membrane blebbing, nuclear condensation, cytoplasmic vacuolization, and cytoplasmic swelling, were observed under fluorescence microscopy, further corroborating the induction of apoptosis by compound **4** and potentially providing evidence for other cell death modes; for example, cytoplasmic or vacuolization is consistent with paraptosis, a noncaspase-dependent programmed cell death.^108^ Further evaluation is required to determine the specific mode of nonapoptotic cell death occurring in HCT-116 and MCF-7 cells, such as investigating MAPK pathway perturbations to assess the involvement of paraptosis or conducting transmission electron microscopy (TEM) analysis to identify features indicative of autophagy. Critically for phenotypic approaches to drug discovery, molecular targets’ deconvolution is essential prior to further preclinical and clinical evaluation. Important in this endeavor are proteolysis targeting chimeras (PROTACs).^116,117^ Technologies including proteomics or kinome studies, to evaluate protein targets or kinase activity perturbation, AI, machine learning, genome mining, and engineering are advancing natural product drug discovery.^118^ Metabolomics analysis, which determines changes in metabolite levels caused by compound treatment, is a crucial step in understanding mechanism(s) of action, revealing alterations in cellular processes, proteins, or molecular targets.^119^ A systematic metabolomics study for alkaloid **4** was undertaken, the findings of which will be reported separately.

The broad-spectrum and potent preclinical in vitro antitumor activity of this newly identified oligocyclotryptamine alkaloid argues for further preclinical evaluation as an experimental and putative antitumor agent; however, its potential noncancer selectivity should be acknowledged, and nanoformulations considered to minimize adverse toxicities.

## Materials and Methods

### Isolation and Characterization of Alkaloids from *Eumachia montana*

#### Plant Material

The leaves of *Eumachia montana* (Blume) I.M.Turner (syn. *Psychotria montana*) were collected in July 2019 from Hulu Langat, Selangor, Malaysia (3.05792° N, 101.87036° E), and the plant identity was determined by Dr K. T. Yong from the Institute of Biological Sciences, Universiti Malaya, Malaysia. A voucher specimen (KLU50139) was deposited at the Herbarium, Universiti Malaya.

#### General Experimental Procedures

Optical rotation values were measured on a JASCO P-1020 automatic digital polarimeter. 1D and 2D NMR spectra were recorded in CDCl_3_ by using tetramethylsilane as an internal standard on a Bruker Avance III 600 MHz NMR spectrometer. HRESIMS data were measured on a Thermo Scientific Q Exactive Plus Hybrid Quadrupole-Orbitrap Mass Spectrometer that was linked to Dionex HPLC systems (compounds **2–4**), and on a Bruker Daltonics micrOTOF-Q III mass spectrometer (compounds **1** and **5**). Semipreparative HPLC was conducted on a Waters Acquity Arc UHPLC System equipped with a Waters 2998 photodiode array (PDA) detector and a Waters Fraction Collector III. X-ray diffraction analysis was carried out on a Rigaku Oxford (formerly Agilent Technologies) SuperNova Dual diffractometer with Cu Kα (λ = 1.54184 Å) radiation at rt.

#### Extraction and Isolation

The dried, ground leaves of *Eumachia montana* (0.98 kg) were extracted exhaustively with 95% EtOH (4 × 10 L) at room temperature. The concentrated extract was added to a 3% tartaric acid solution with vigorous stirring. The acidified solution was then filtered through kieselguhr to remove the insoluble material. Concentrated NH_3_ solution was added gradually to basify the filtrate up to pH 10. The basified filtrate was extracted exhaustively with CHCl_3_, washed with water, dried over anhydrous Na_2_SO_4_, and concentrated in vacuo to yield 15.2 g of a crude alkaloid mixture. The crude alkaloid mixture was fractionated initially by vacuum-column chromatography over silica gel with gradient elution (CHCl_3_/MeOH, 1:0 → 7:3) to afford nine major fractions (PML-A–PML-I). PML-C (365 mg) was subjected to centrifugal preparative thin-layer chromatography (CTLC) (silica gel, CHCl_3_/Hex/NH_3_) to yield compound **1** (135 mg). PML-D (4.43 g) was rechromatographed using vacuum-column chromatography (silica gel, EA/MeOH, with increasing percentages of MeOH) to give nine subfractions (PML-D-A–PML-D-I). Fractionation of PML-D-A (349 mg), PML-D-B (2.18g) and PML-D-C (417 mg) with CTLC (silica gel, CHCl_3_/MeOH/NH_3_) afforded partially separated subfractions which were then purified by semipreparative reversed-phase HPLC (Waters XBridge BEH C_18_ OBD prep column, 5 μm, 10 × 100 mm) under isocratic elution [MeOH(85%): H_2_O­(15%) (0.1% NH_3_), 3.0 mL/min for 40 min] at 40 °C to yield alkaloid **2** (82 mg), **3** (98 mg) and **4** (58 mg).

#### Structure Elucidation

Variable-temperature NMR spectroscopy was performed on compounds **2–4** at room temperature (25 °C), high temperature (55 °C), and extremely low temperature (−32 °C) to obtain detailed NMR spectra. HRESIMS analysis of compounds **1–5** determined their molecular weights and chemical formulas. Electronic circular dichroism (ECD) measurements for compounds **2–4** provided additional structural confirmation. The structure of compound **1** was determined by X-ray diffraction and ^1^H NMR spectroscopy, while compound **5** was characterized through HRESIMS and a comprehensive set of NMR experiments, including ^13^C, ^1^H, HMBC, COSY, and NOESY.

#### (−)-Calycanthine (**1**)

Light yellowish crystals; [α]_D_ + 106 (*c* 0.43, EtOH); ^1^H NMR data (CDCl_3_, 600 MHz) δ 7.00 (dd, *J* = 7.8, 1.5 Hz, 2H), 6.81 (t, *J* = 7.6 Hz, 2H), 6.53 (td, *J* = 7.5, 1.2 Hz, 1H), 6.26 (dd, *J* = 8.0, 1.2 Hz, 2H), 4.57 (s, 2H), 4.30 (s, 2H), 3.11 (td, *J* = 13.2, 5.5 Hz, 2H), 2.61 (ddd, *J* = 11.5, 5.5, 1.6 Hz, 2H), 2.40 (s, 6H), 2.25 (ddd, *J* = 13.1, I1.6, 4.1 Hz, 2H), 1.28 (ddd, *J* = 13.2, 4.1, 1.6 Hz, 2H); HRESIMS: *m*/*z* 347.2250 [M + H]^+^ (calcd for C_22_H_26_N_4_ + H^+^, 347.2230).

#### Oleoidine (**2**)

Light yellowish powder; [α]_D_ + 106 (*c* 0.43, EtOH): ^13^C NMR data (CD_3_OD, 150 MHz, 328 K) δ 150.75, 150.68, 148.08, 147.69, 133.20, 132.89, 132.29, 131.42, 128.15, 127.60, 125.36, 125.25, 124.80, 124.52, 124.46, 124.37, 123.87, 122.71, 122.32, 121.94, 121.76, 119.11, 118.29, 118.17, 117.22, 108.27, 108.10, 86.33, 86.29, 85.21, 84.20, 82.58, 81.89, 62.61, 62.31, 61.04, 60.53, 60.37, 59.88, 59.62, 52.49, 51.99, 51.87, 51.72, 50.95, 50.89, 48.43, 38.13, 37.86, 37.36, 37.19, 35.19, 35.05, 34.99, 34.74, and 34.31; HRESIMS: *m*/*z* 1035.6217 [M + H]^+^ (calcd for C_66_H_74_N_12_ + H^+^, 1035.6232).

#### Caledonine (**3**)

Light yellowish powder; [α]_D_ + 139 (*c* 0.80, EtOH); ^13^C NMR data (CD_3_OD, 150 MHz, 328 K) δ 150.18, 149.84, 147.62, 147.46, 147.02, 132.57, 132.11, 131.38, 130.75, 127.40, 127.32, 124.57, 124.38, 124.09, 123.74, 123.14, 122.42, 121.86, 121.67, 121.04, 117.72, 117.37, 107.34, 107.07, 85.58, 85.22, 84.31, 83.45, 61.79, 59.82, 59.68, 59.23, 59.16, 59.05, 58.94, 51.58, 51.15, 50.81, 50.66, 50.17, 47.73, 37.88, 37.56, 37.19, 36.93, 36.74, 34.46, 34.36, 34.29, 34.14, and 34.01; HRESIMS: *m*/*z* 1207.7219 [M + H]^+^ (calcd for C_77_H_86_N_14_ + H^+^, 1207.7233).

#### Eumatanine (**4**)

Colorless oil; [α]_D_ + 122 (*c* 0.33, EtOH); ^13^C NMR data (CD_3_OD, 150 MHz, 328 K) δ 150.87, 150.67, 148.62, 148.18, 147.88, 133.33, 133.29, 132.91, 132.16, 131.52, 128.12, 127.79, 125.32, 125.30, 124.95, 124.84, 124.63, 124.49, 124.44, 123.83, 123.58, 123.31, 122.66, 122.32, 121.93, 121.76, 119.08, 118.18, 118.12, 118.08, 117.68, 108.13, 108.07, 86.34, 86.27, 85.72, 85.12, 84.19, 82.57, 81.83, 62.61, 60.57, 59.90, 59.81, 59.66, 52.44, 51.88, 51.49, 50.91, 48.45, 38.63, 38.25, 38.03, 37.48, 37.41, 35.10, 35.05, 34.86, 34.77, 34.75; HRESIMS: *m*/*z* 1379.8215 [M + H]^+^ (calcd for C_88_H_98_N_16_ + H^+^, 1379.8233).

#### Eumatricine (**5**)

Light yellowish powder; ^1^H NMR (CDCl_3_, 600 MHz) and ^13^C NMR (CDCl_3_, 150 MHz) data, see Table 1; HRESIMS: *m*/*z* 183.0932 [M + H]^+^ (calcd for C_12_H_10_N_2_ + H^+^, 183.0917).

### Cell Culture Assays

#### General Cell Culture

The cell lines used in this study include 7 human epithelial cancer (carcinoma) cell lines including HCT-116, HT-29 colorectal, MCF-7, MDA-MB-468 breast, A549 nonsmall cell lung (NSCL), U373 V­(vector control), U373 M (methylguanine DNA-methyltransferase (MGMT)-transfected) glioblastoma multiforme (GBM) and one nontransformed fetal lung fibroblast line (MRC-5), U373 V and U373 M isogenic cell lines were a gift from Schering Plough corporation. All others were originally purchased from the European Collection of Cell Culture (supplied via Sigma-Aldrich) and American Type Tissue Culture Collection (ATCC). All cell lines were verified as mycoplasma-free and stored in liquid nitrogen (−196 °C). After reviving frozen cells, each cell line was passaged for ∼4 months before being discarded to minimize genotypic drift as a result of continual passage.

All cell lines were serially subcultured in 25 cm^2^ tissue culture flasks once or twice weekly, depending on the cell type, incubating at 37 °C in a humidified atmosphere of 95% air and 5% CO_2_ in a LEEC incubator. To maintain normal logarithmic cell growth, cells were 70–80% confluent at the time of subculturing or setting up experiments.

MCF-7, HCT-116, and HT-29 cell lines were subcultured in Roswell Park Memorial Institute (RPMI)-1640 medium supplemented with 10% v/v fetal bovine serum (FBS). For MRC-5 fibroblast medium consisted of minimum essential medium (MEM) supplemented with 1% 0.1 mM nonessential amino acids (NEAA), 1% 1 M N-2-hydroxyethylpiperazine-N’-2-ethanesulfonic acid (HEPES), 1% 200 mM l-glutamine, 1% penicillin-streptomycin, and 10% v/v FBS. For MDA-MB-468 medium comprised MEM supplemented with 1% 200 mM l-glutamine and 1% penicillin-streptomycin, as well as 10% v/v FBS. U373 V and U373 M were subcultured in RPMI 1640 medium, to which 1% mM NEAA, 1% gentamicin (50 μg/mL), 1% G418 sulfate (400 μg/mL), 1% 200 mM l-glutamine, and 10% v/v FBS were added.

#### Compound Stocks Preparation

To obtain compound stocks that apply afterward in cell culture assays, 10 mg/mL compounds **2–4** (equating to 0.966, 0.828, and 0.7247 mM compounds **2**, **3**, and **4**, respectively) in dimethylsulfoxide (DMSO) were prepared, aliquoted, and stored at −80 °C.

#### MTT Assay

3-[4,5-Dimethylthiazol-2-yl]-2,5-diphenyl tetrazolium bromide (MTT) assays were used to assess the ability of test compounds to inhibit cell growth and/or evoke cytotoxicity.^112^ Cells (2.(5–5) × 10^3^ cells/well) were seeded in 96-well plates, and time zero (T0) plates were set for each cell line for every independent trial. MTT assays were performed at the time of test agent addition (T0) and following 72 h exposure of cells to compounds **2**–**4**.^113^ The absorbance was measured (a surrogate marker for the number of viable cells) at 570 nm using a PerkinElmer Envision plate reader. Data were collected and analyzed using Wallac Envision Manager version 1.12 software.

Means and standard deviations (SDs) of repeats (*n* = 6) were calculated, and the optical density (OD)_570_ for estimated GI_50_ (the dose that inhibits the growth of cells by 50%) values were calculated using the following formula:

$$((\mathrm{control}\;{\mathrm{OD}}_{570}-\mathrm{initial}\;{\mathrm{OD}}_{570})+\mathrm{initial}\;{\mathrm{OD}}_{570})/2={\mathrm{OD}}_{570}\;\mathrm{at}\;{\mathrm{GI}}_{50}$$

Estimated GI_50_ values were estimated by interpolation. Independent MTT assays for each trial were repeated at least 3 times, and mean ± SEM GI_50_ values were calculated.

#### Cell Count Assay

To validate the results from the MTT assay, cell counting assays were conducted, as this assay directly counts the number of viable cells. Cells (2 × 10^4^ cells/well) were seeded in 6-well plates, and a separate T0 plate was seeded for each cell line for every individual trial. Cells were counted on a hemocytometer on the same day of drug treatment (T0 plates) and following 72 h exposure of cells to test compounds. For experimental treatment plates, cells were exposed to medium with final concentrations equivalent to 1 × GI_50_ and 2 × GI_50_ (the GI_50_ values were obtained from the corresponding MTT assays) of test compounds **2–4.**

#### Clonogenic Assay

The clonogenic assay is able to detect the capability of a single cell to survive and retain proliferative capacity following a brief exposure (e.g., 24 h) to test compounds.^40^ Cells (250 cells/well) were seeded in 6-well plates and were exposed to test compounds **2–4** for 24 h at concentrations of 1 × GI_50_ and 2 × GI_50_ values (obtained from the corresponding MTT assays). Thereafter, cells were allowed to form colonies in an incubator at 37 °C for 7–14 days. The colonies were fixed (100% methanol, 10 min), washed, and stained using methylene blue (0.05%), then were counted when colonies in control wells contained ≥50 cells.

#### Cell Cycle Analysis

In this study, the cell cycle is analyzed by flow cytometry, and the DNA intercalating stain PI is used to bind cellular DNA.^114^ Cells ((1–2) × 10^5^, 2 mL medium) were seeded in 6-well plates (overnight at 37 °C) and were exposed to the tested compound **4** for 24 and 72 h at concentrations of 1 × GI_50_ and 2 × GI_50_ values. Subsequently, cells were harvested and centrifuged (1350 rpm, 4 °C, 7 min), before the cell pellets were resuspended in 300–500 μL hypotonic fluorochrome solution (50 μg/mL propidium iodide (PI); 0.1 mg/mL Ribonuclease A (RNA); 0.1 v/v Triton X – 100; 0.1 w/v sodium citrate).^114^ Samples in hypotonic fluorochrome solution were kept at 4 °C in the dark for ≥1 h before being processed on a ID7000 Sony flow cytometer, and the acquired data were analyzed using Weasel software (≥3 independent trials performed, where *n* = 2, >10,000 events recorded per sample).

#### Apoptosis Assay

In this study, the FITC annexin V is used in conjunction with propidium iodide (PI), which is a DNA-binding dye that permeates the membranes of dead or damaged cells but not viable cells with intact membranes. Thus, this method allows the identification of early apoptotic cells (PI negative, FITC Annexin V positive), late-stage apoptotic cells (PI positive, FITC Annexin V positive), as well as viable cells (PI negative, FITC Annexin V negative).^115^ Invitrogen eBioscience FITC Annexin V Apoptosis Detection Kits (containing 10× annexin V binding buffer, FITC annexin V, and PI staining solution) were used for apoptosis assays.

Cells ((1–2) × 10^5^, 2 mL medium) were seeded in 6-well plates (overnight at 37 °C) and were exposed to compound **4** for 24 and 72 h at concentrations of 1 × GI_50_ and 2 × GI_50_ values. Subsequently, cells were harvested and centrifuged (1350 rpm, 4 °C, 7 min), before the cell pellets were incubated in annexin-V-FITC (5 μL) plus 100 μL 1 × annexin-V buffer for 15 min in the dark on ice. PI (10 μL; 50 μg/mL in phosphate-buffered saline (PBS)) plus 400 μL annexin-V buffer were then added to cells, samples were subsequently incubated on ice for ≥ 10 min in the dark before flow cytometry analysis (ID7000 Sony flow cytometer), ≥3 independent trials performed where *n* = 2, >10,000 events recorded per sample. The acquired data were analyzed on FlowJo software.

#### Caspase-3/7 Activity Assay and Detection of Reactive Oxygen Species (ROS)

Cells ((3–5) × 10^3^) were seeded per well in 96-well opaque white culture plates (overnight at 37 °C). Cells were exposed to compound **4** at a concentration of 1 × GI_50_ value for 48 h for caspase-3/7 assay (≥3 independent trials where *n* = 3) and 24 h for ROS assay (≥3 independent trials where *n* = 2). According to the manufacturer’s protocol, the Caspase-Glo 3/7 Assay (Promega) was used to detect caspase 3/7 activity of tested cells, and the ROS-Glo^T^ H_2_O_2_ Assay (Promega) was used to determine ROS production. Well luminescence was recorded by using a PerkinElmer Envision plate reader.

#### γH2AX Assay

Cells were seeded in 6-well plates ((1–2) × 10^5^ cells/well, 2 mL medium) or 10 cm Petri dishes (1 × 10^6^ cells/well, 10 mL medium) and allowed to attach overnight before introduction of compound **4** (24 and 72 h) at concentrations of 1 × GI_50_ and 2 × GI_50_ values. Subsequently, cells were harvested and centrifuged (1250 rpm, 10 min, room temperature (rt)), before the retained cell pellets were fixed (1% methanol-free formaldehyde in PBS, 5 min, rt), permeabilized (Triton X-100 in PBS, 1 min, rt), blocked (1% FBS in PBS, 30 min, rt), and incubated with primary antibody anti-γH2AX (Millipore, 1:3333 dilution in 1% FBS in PBS, 1.5 h, rt) and secondary antibody Alexa Flour 488 (Invitrogen, 1:1750 dilution in 1%FBS in PBS, 1 h, rt). For concurrent cell cycle analysis, cells were resuspended in 300 μL PI (50 μg/mL and RNA 100 μg/mL in PBS) solution and incubated for >10 min at rt prior to analysis on a flow cytometer (ID7000 Sony flow cytometer), ≥ 3 independent trials performed where *n* = 2, >10,000 events recorded per sample. The acquired data were analyzed on FlowJo software.

#### Fluorescent Microscopy

Cells (3000–5000 cells/well) were seeded into 96-Well Glass-Bottom CELLview Microplates (Greiner) and were allowed to attach overnight at 37 °C. Following 24 or 72 h exposure to compound **4** at concentrations of 0.5 × GI_50_ and 1.0 × GI_50_, cells were fixed (3.7% formaldehyde in PBS, 15 min, rt), permeabilized (PBT (0.1% Triton X-100 in PBS), 2 min, rt), blocked (PBT + 1% bovine serum albumin (BSA), 1 h, rt), and finally incubated with fluorescence antibodies separately. Antibodies used in this study include Alexa Fluor 488 phalloidin (Invitrogen, 1:160,000 dilution in PBS, 45 min, rt) and DRAQ5 fluorescent probe solution (5 mM) (Thermo-Scientific, 1:1000 dilution in PBS, 2–5 min in the dark, 37 °C). Cells were kept in the fridge in PBS before being observed under an automated microscope (ZEISS, Celldiscoverer 7 (LSM900, confocal)).

#### Statistical Analyses

Unless otherwise stated, independent repeat experiments were performed ≥3 × where internal replicates ≥2 (with representative experiments illustrated). One-way and two-way analyses of variance (ANOVAs) were used to determine statistical significance. Dunnett’s multiple comparison tests were used to determine the minimal level of significance (*p* < 0.05).

## Supplementary Material



## Notes

Figure S1. ^1^H NMR spectrum of (−)-calycanthine (**1**); Figure S2. X-ray crystal structure of (−)-calycanthine (**1**); Figure S3. ^1^H NMR spectrum of oleoidine (**2**) at rt in CDCl_3_; Figure S4. ^1^H NMR spectra of oleoidine (**2**) at 243 and 298 K in CD_3_OD; Figure S5. ^1^H NMR spectra of oleoidine (**2**) at 328 and 298 K in CD_3_OD; Figure S6. ^13^C NMR spectrum of oleoidine (**2**) at 328 K in CD_3_OD; Figure S7. ^1^H NMR spectrum of caledonine (**3**) at rt in CDCl_3_ Figure S8. ^1^H NMR spectrum of caledonine (**3**) at rt in CD_3_OD; Figure S9 ^1^H NMR spectra of caledonine (**3**) at 241 and 298 K in CD_3_OD; Figure S10. ^1^H NMR spectra of caledonine (**3**) at 328 and 298 K in CD_3_OD; Figure S11. ^13^C NMR spectrum of caledonine (**3**) at 328 K in CD_3_OD; Figure S12. Mass spectra of oleoidine (**2**) and caledonine (**3**); Figure S13. ECD spectra of oleoidine (**2**), caledonine (**3**), and eumatanine (**4**); Figure S14. ^1^H NMR spectrum of eumatanine (**4**) at rt in CDCl_3_; Figure S15. ^1^H NMR spectra of eumatanine **(4)** at 243 and 298 K in CD_3_OD; Figure S16. ^1^H NMR spectra of eumatanine **(4)** at 328 and 298 K in CD_3_OD; Figure S17. ^13^C NMR spectrum of eumatanine **(4)** at 328 K in CD_3_OD; Figure S18. mass spectrum of eumatanine (**4**); Figure S19. ^1^H NMR spectrum of eumatricine (**5**); Figure S20. ^13^C NMR spectrum of eumatricine (**5**); Figure S21. COSY spectrum of eumatricine (**5**); Figure S22. HSQC spectrum of eumatricine (**5**); Figure S23. HMBC spectrum of eumatricine (**5**); Figure S24. NOESY spectrum of eumatricine (**5**); and Figure S25. Mass spectrum of eumatricine (**5**) (PDF)

## Abbreviations

MTT: 3-[4,5-Dimethylthiazol-2-yl]-2,5-diphenyl tetrazolium bromide
ROS: reactive oxygen species
NPs: Natural Products
NMR: nuclear magnetic resonance
ECD: electronic circular dichroism
HRESIMS: high-resolution electrospray ionization mass spectrometry
GBM: glioblastoma multiforme
MGMT: methylguanine DNA-methyltransferase
O6MG: O6 methyl guanine
ADCs: antibody-drug conjugates
EPR: enhanced permeation and retention
RES: reticuloendothelial system
Aft: Apoferritin
Pgp: P-glycoprotein
MDR: multidrug resistance
RB: retinoblastoma
CDKs: cyclin-dependent kinases
DHFR: dihydrofolate reductase
MCM3/5: minichromosome maintenance complex component 3 and 5
PI: propidium iodide
B-CLL: B-cell chronic lymphocytic leukemia
AMPK: adenosine monophosphate-activated protein kinase
SPD: styrylpyrone derivative
ER: endoplasmic reticulum
MOMP: mitochondrial outer membrane permeabilization
XIAP: X-linked inhibitor of apoptosis
FADD: FAS-associated death domain
DISC: death-inducing signaling complex
Cyt c: cytochrome *c*
IMS: intermembrane space
NK: natural killer
NP: natural product
DSB: double-strand breaks
NCCD: the nomenclature committee on cell death
MAPKs: mitogen-activated protein kinases
TEM: transmission electron microscopy
TLC: thin layer chromatography
CC: column chromatography
CTLC: centrifugal thin layer chromatography
HPLC: high-performance liquid chromatography
TMS: tetramethylsilane
DMSO: dimethylsulfoxide
NSCL: nonsmall cell lung
ATCC: American Type Tissue Culture Collection
RPMI: Rosewell Park Memorial Institute
FBS: fetal bovine serum
MEM: minimum essential medium
NEAA: nonessential amino acids
SDs: standard derivations
OD: optical density
RNA: Ribonuclease A
PBS: phosphate-buffered saline
BSA: bovine serum albumin
