# Supplementary material for: Isolation, Characterization, and Anticancer Evaluation of Alkaloids from Eumachia montana (Rubiaceae)
Source: ACS Omega. 2025 Aug 4;10(32):35719–37. doi: 10.1021/acsomega.5c02401 (PMC12368673; doi:10.1021/acsomega.5c02401)
Supplement: Supplementary file 1 [file ao5c02401_si_001.pdf]

Supporting Information

Isolation, Characterization and Anticancer Evaluation of Alkaloids from *Eumachia montana* (Rubiaceae)

Yuye Shuai[1,2], *Dong-hyun Kim*[1], *Kuan-Hon Lim*[2], *Premanand Krishnan*[2], *Yun-Yee Low*,[3] *Kien-Thai Yong*,[4] *Tracey D. Bradshaw*[1*]

[1]School of Pharmacy, Biodiscovery Institute, The University of Nottingham, University Park, Nottingham, Nottinghamshire NG72ND, U.K.

[2]School of Pharmacy, Faculty of Science and Engineering, University of Nottingham Malaysia, 43500 Semenyih, Selangor, Malaysia

[3]Department of Chemistry, Faculty of Science, Universiti Malaya, 50603, Kuala Lumpur, Malaysia

[4]Institute of Biological Sciences, Faculty of Science, Universiti Malaya, 50603, Kuala Lumpur, Malaysia

Table of Contents

No.		Pages
1	Figure S1. ^{1}H NMR Spectrum of (−)-Calycanthine (**1**)	3
2	Figure S2. X-ray Crystal Structure of (−)-Calycanthine (**1**)	4-5
3	Figure S3. ^{1}H NMR Spectrum of Oleoidine (**2**) at rt in $CDCl_3$	6
4	Figure S4. ^{1}H NMR Spectra of Oleoidine (**2**) at 243 K and 298 K in CD_3OD	7
5	Figure S5. ^{1}H NMR Spectra of Oleoidine (**2**) at 328 K and 298 K in CD_3OD	8
6	Figure S6. ^{13}C NMR Spectrum of Oleoidine (**2**) at 328 K in CD_3OD	9
7	Figure S7. ^{1}H NMR Spectrum of Caledonine (**3**) at rt in $CDCl_3$	10
8	Figure S8. ^{1}H NMR Spectrum of Caledonine (**3**) at rt in CD_3OD	11
9	Figure S9. ^{1}H NMR Spectra of Caledonine (**3**) at 241 K and 298 K in CD_3OD	12
10	Figure S10. ^{1}H NMR Spectra of Caledonine (**3**) at 328 K and 298 K in CD_3OD	13
11	Figure S11. ^{13}C NMR Spectrum of Caledonine (**3**) at 328 K in CD_3OD	14
12	Figure S12. Mass Spectra of Oleoidine (**2**) and Caledonine (**3**)	15
13	Figure S13**.** ECD spectra of Oleoidine (**2**), Caledonine (**3**), and Eumatanine (**4**)	16
14	Figure S14. ^{1}H NMR Spectrum of Eumatanine (**4**) at rt in $CDCl_3$	17
15	Figure S15. ^{1}H NMR Spectra of Eumatanine **(4)** at 243 K and 298 K in CD_3OD	18
16	Figure S16. ^{1}H NMR Spectra of Eumatanine **(4)** at 328 K and 298 K in CD_3OD	19
17	Figure S17. ^{13}C NMR Spectrum of Eumatanine **(4)** at 328 K in CD_3OD	20
18	Figure S18. Mass Spectrum of Eumatanine (**4**)	21
19	Figure S19. ^{1}H NMR Spectrum of Eumatricine (**5**)	22
20	Figure S20. ^{13}C NMR Spectrum of Eumatricine (**5**)	23
21	Figure S21. COSY Spectrum of Eumatricine (**5**)	24
22	Figure S22. HSQC Spectrum of Eumatricine (**5**)	25
23	Figure S23. HMBC Spectrum of Eumatricine (**5**)	26
24	Figure S24. NOESY Spectrum of Eumatricine (**5**)	27
25	Figure S25. Mass Spectrum of Eumatricine (**5**)	28

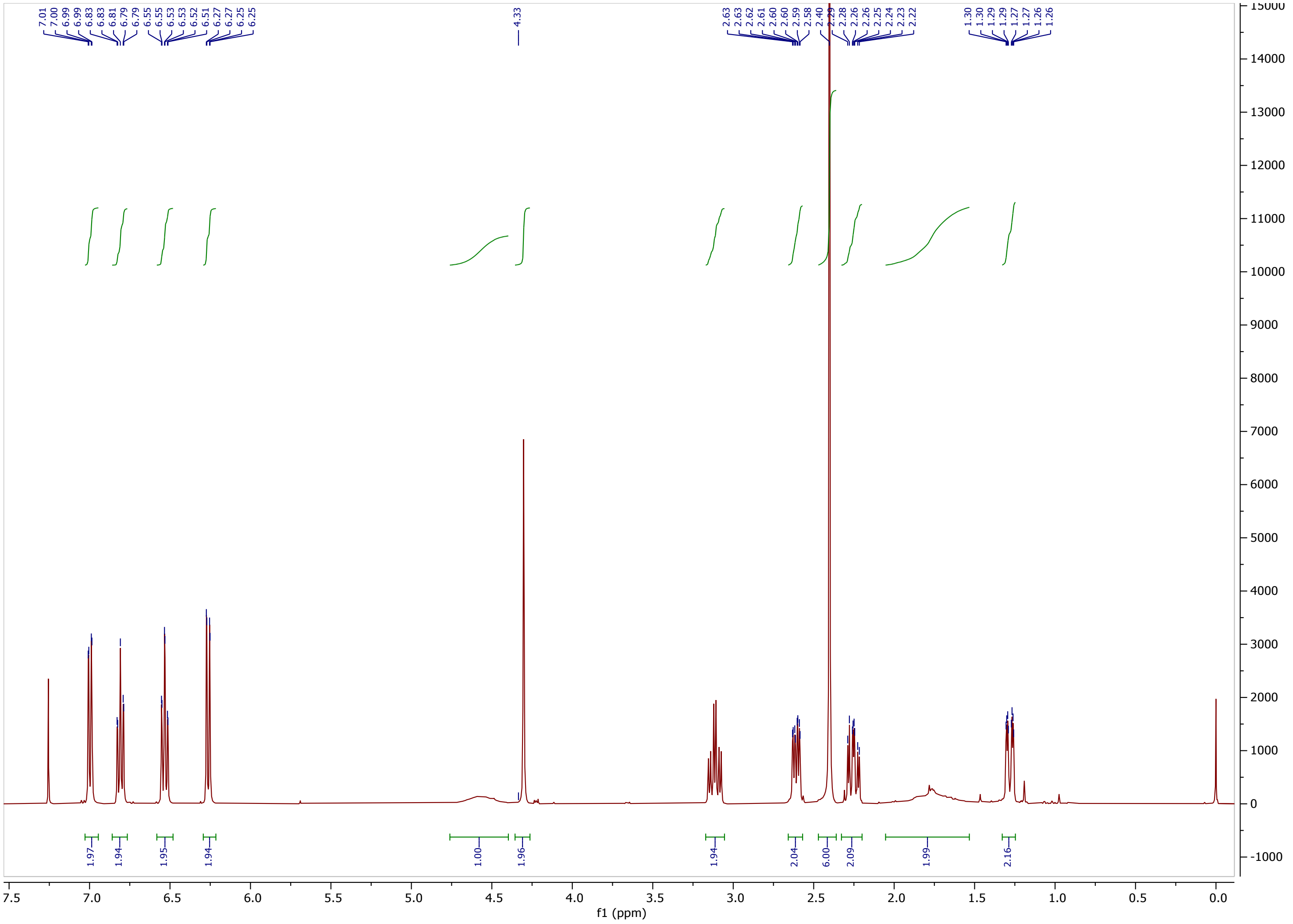

Figure S1. ^{1}H NMR Spectrum of (−)-Calycanthine (**1**)

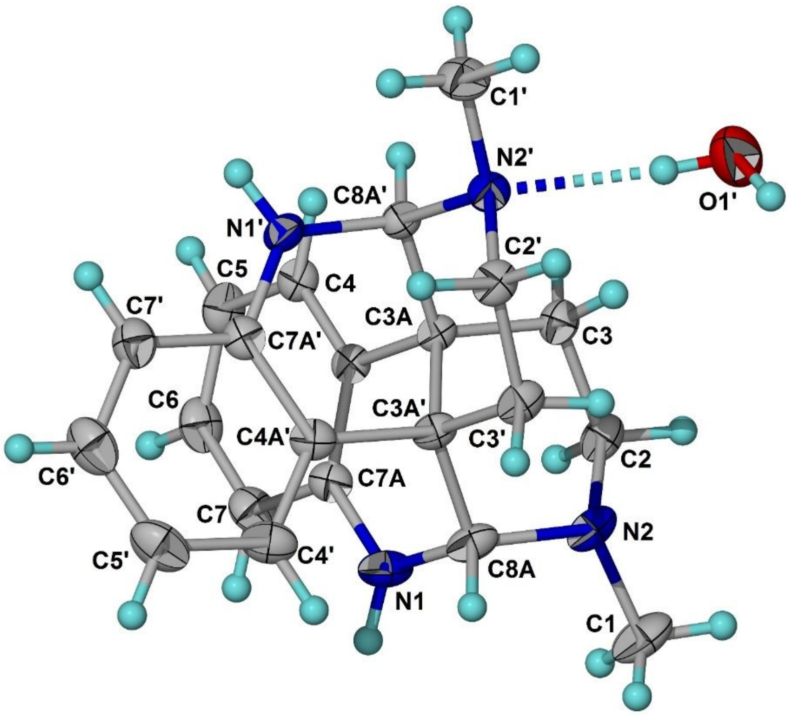

Figure S2. X-ray Crystal Structure of (−)-Calycanthine (**1**)

Crystallographic data of (−)-Calycanthine (**1**): Colorless block crystals, $C_{22}H_{26}N_4.H_2O$, Mr = 364.48, orthorhombic, space group $P2_12_12_1$, a = 10.43715(15) Å, b = 12.9710(2) Å, c = 13.8569(2) Å, V = 1875.95(5) Å^3, Z = 4, D_{calcd} = 1.291 gcm^{-3}, crystal size 0.5 x 0.5 x 0.2 mm^3, $F(000)$ = 784, Cu Kα radiation (λ = 1.54184 Å), T = 293(2) K, 4650 reflections measured (9.34° ≤ 2θ ≤ 147.878°), 3212 unique (R_{int} = 0.0161, R_{sigma} = 0.0233) which were used in all calculations. The final R_1 value was 0.0431 [$I>2\sigma(I)$] and wR_2 was 0.1123.

Crystal data and structure refinement for (−)-Calycanthine (**1**)	
Molecular formula	$C_{22}H_{26}N_4.H_2O$
Molecular weight, M_r	364.48
Temperature during diffraction experiment, T	293(2) K
X-ray source	Cu K_α(λ = 1.54184)
Crystal system	Orthorhombic
Space group	$P2_12_12_1$
a	10.43715(15) Å
b	12.9710(2) Å
c	13.8569(2) Å
α	90°
β	90°
γ	90°
Volume, V	1875.95(5) Å^3
No. of molecule per unit cell, Z	4
Density (calcd)	1.291 g/cm^3
μ	0.638 mm^{-1}
F(000)	784
Crystal size	0.5 × 0.5 × 0.2 mm
2θ range for data collection	9.34 to 147.878°
Index ranges	$-12 \leq h \leq 12$, $-15 \leq k \leq 15$, $-16 \leq l \leq 16$
Reflections collected	4650
Independent reflections	3212 [R_{int} = 0.0161, R_{sigma} = 0.0233]
Data/restraints/parameters	3212/0/249
Goodness-of-fit on F^2	1.073
Final R indexes [$I \geq 2\sigma(I)$]	R_1 = 0.0431, wR_2 = 0.1101
Final R indexes [all data]	R_1 = 0.0449, wR_2 = 0.1123
Largest diff. peak/hole / e Å^{-3}	0.17/–0.31

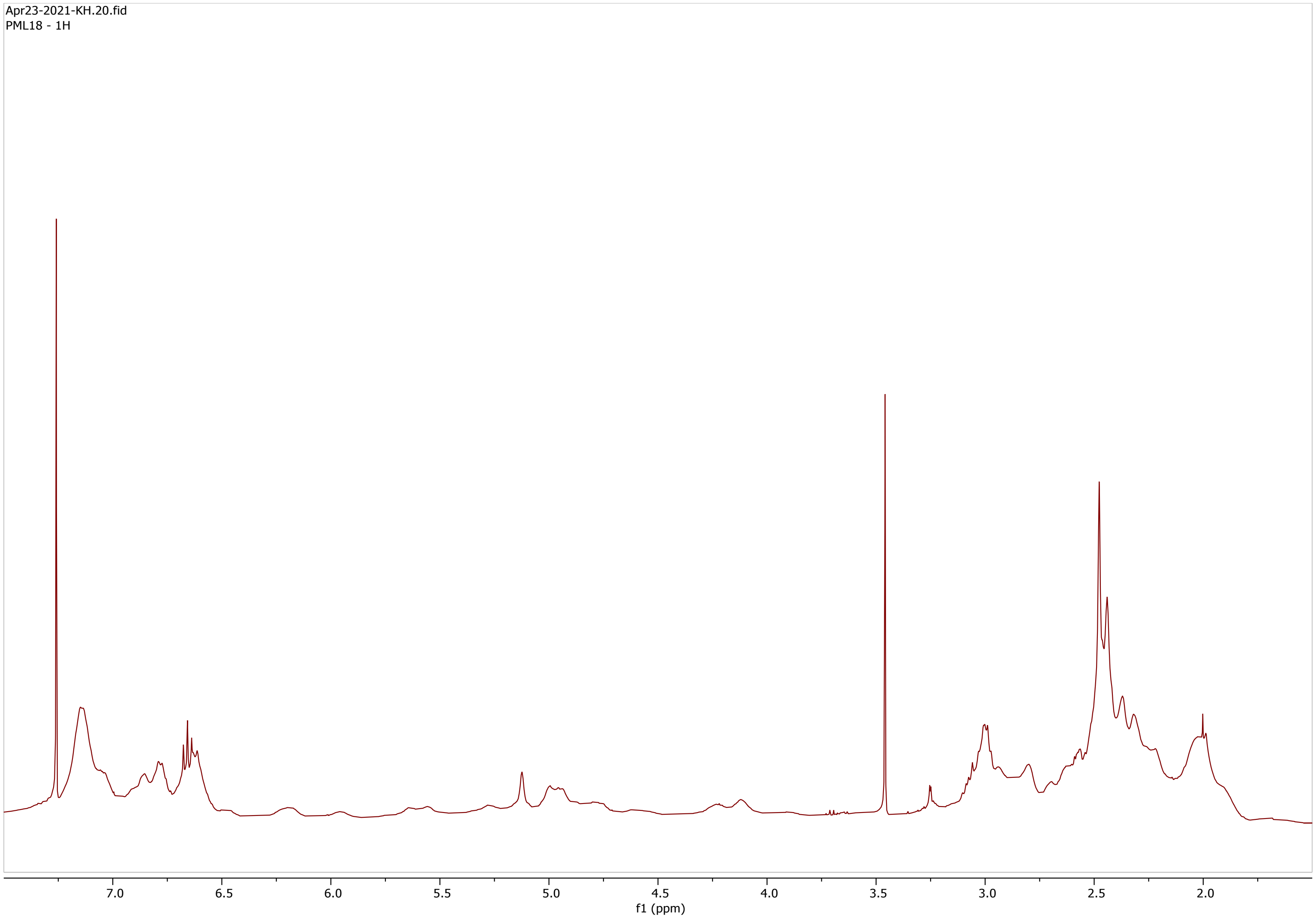

Figure S3. ^{1}H NMR Spectrum of Oleoidine (**2**) at rt in $CDCl_3$

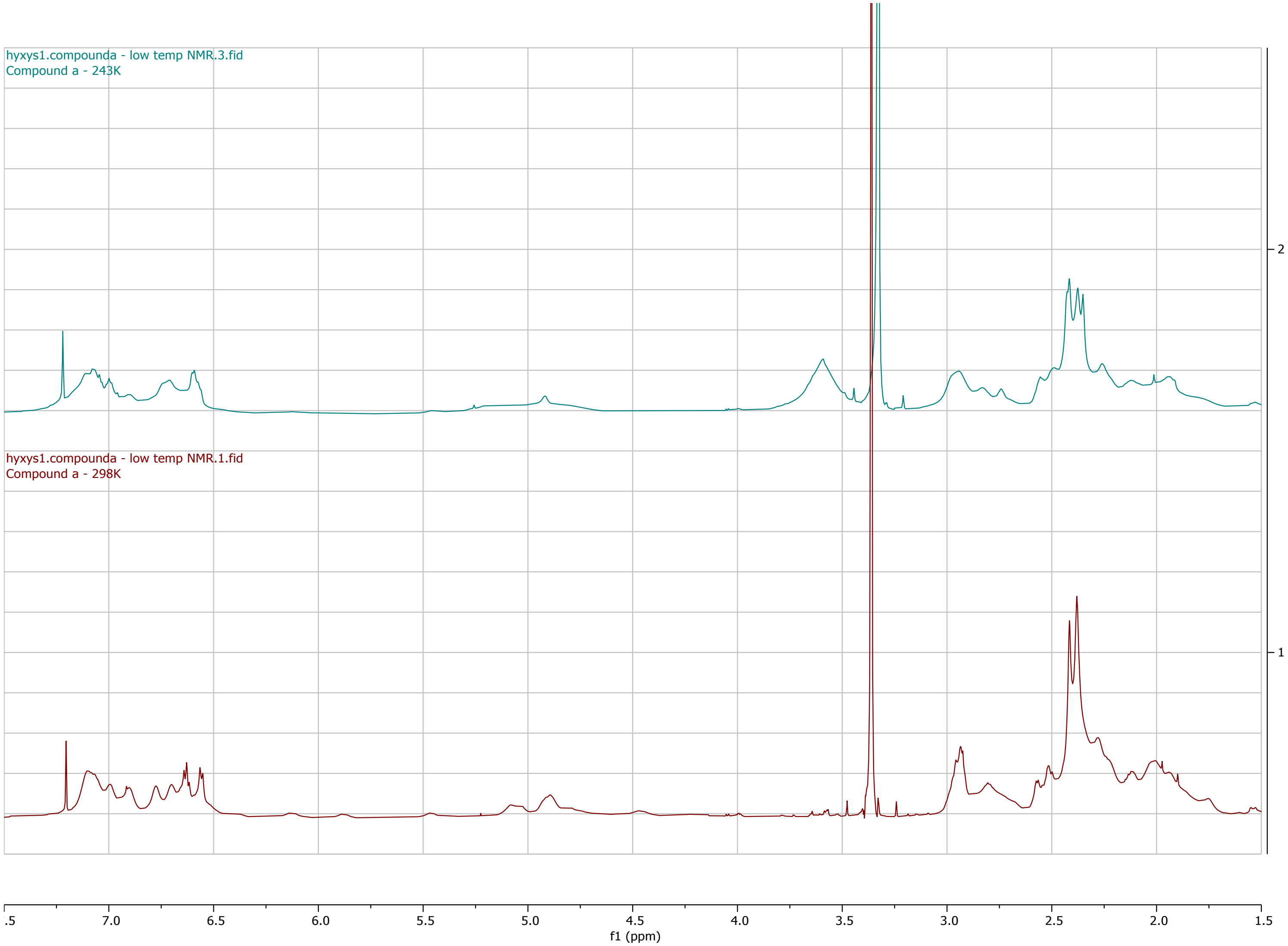

Figure S4. ^{1}H NMR Spectra of Oleoidine (**2**) at 243 K and 298 K in CD_3OD

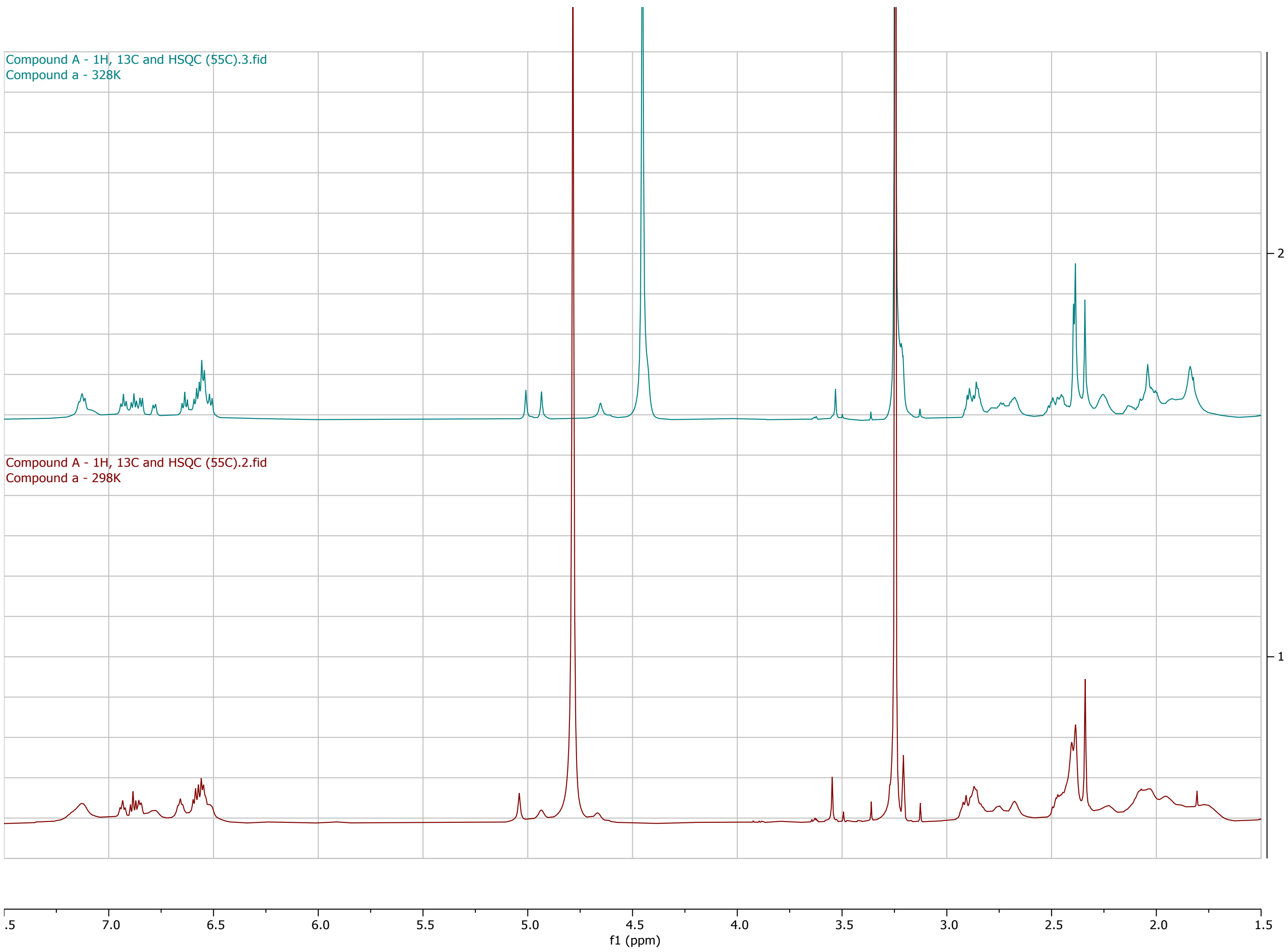

Figure S5. ^{1}H NMR Spectra of Oleoidine (**2**) at 328 K and 298 K in CD_3OD

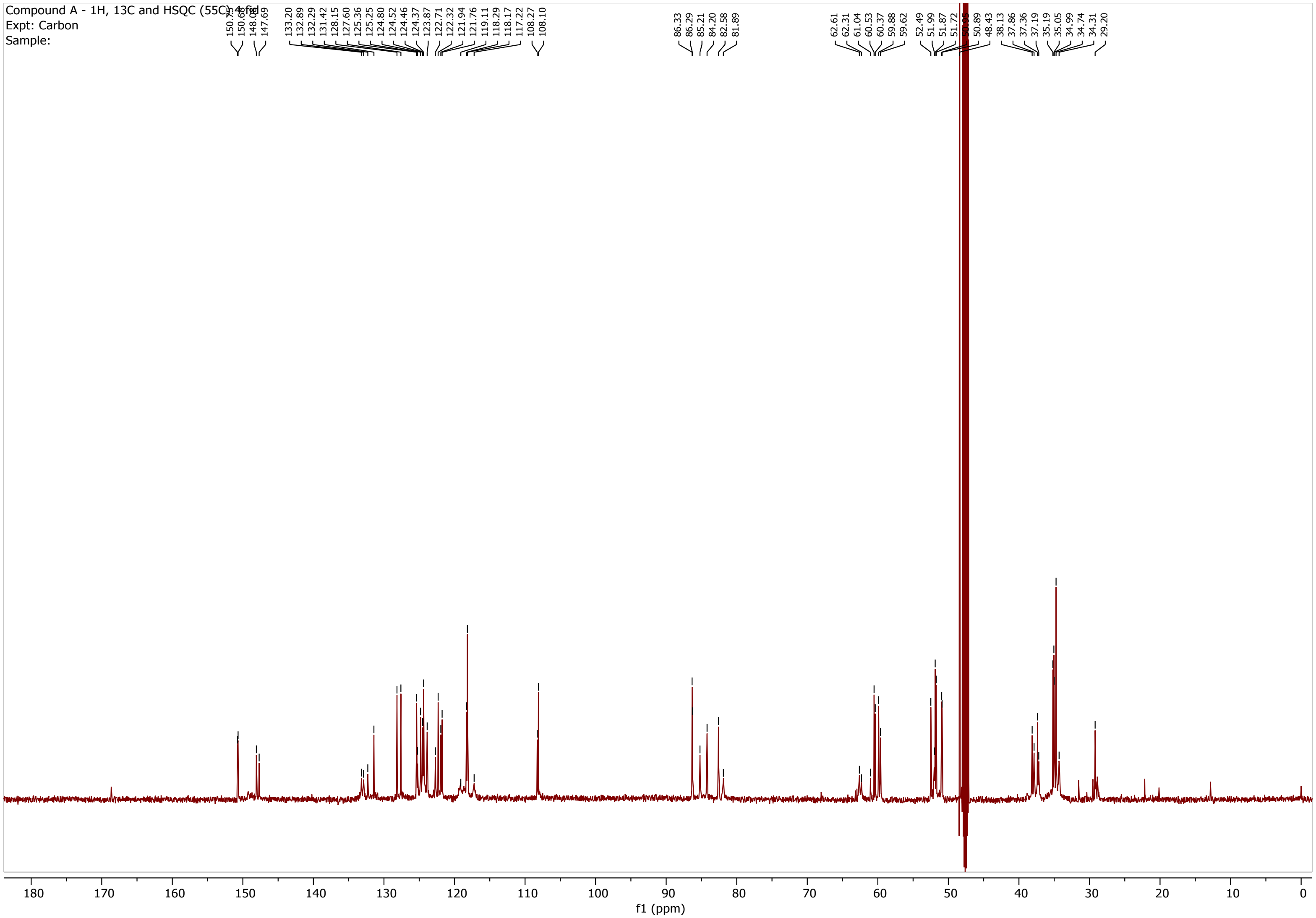

Figure S6. ^{13}C NMR Spectrum of Oleoidine (**2**) at 328 K in CD_3OD

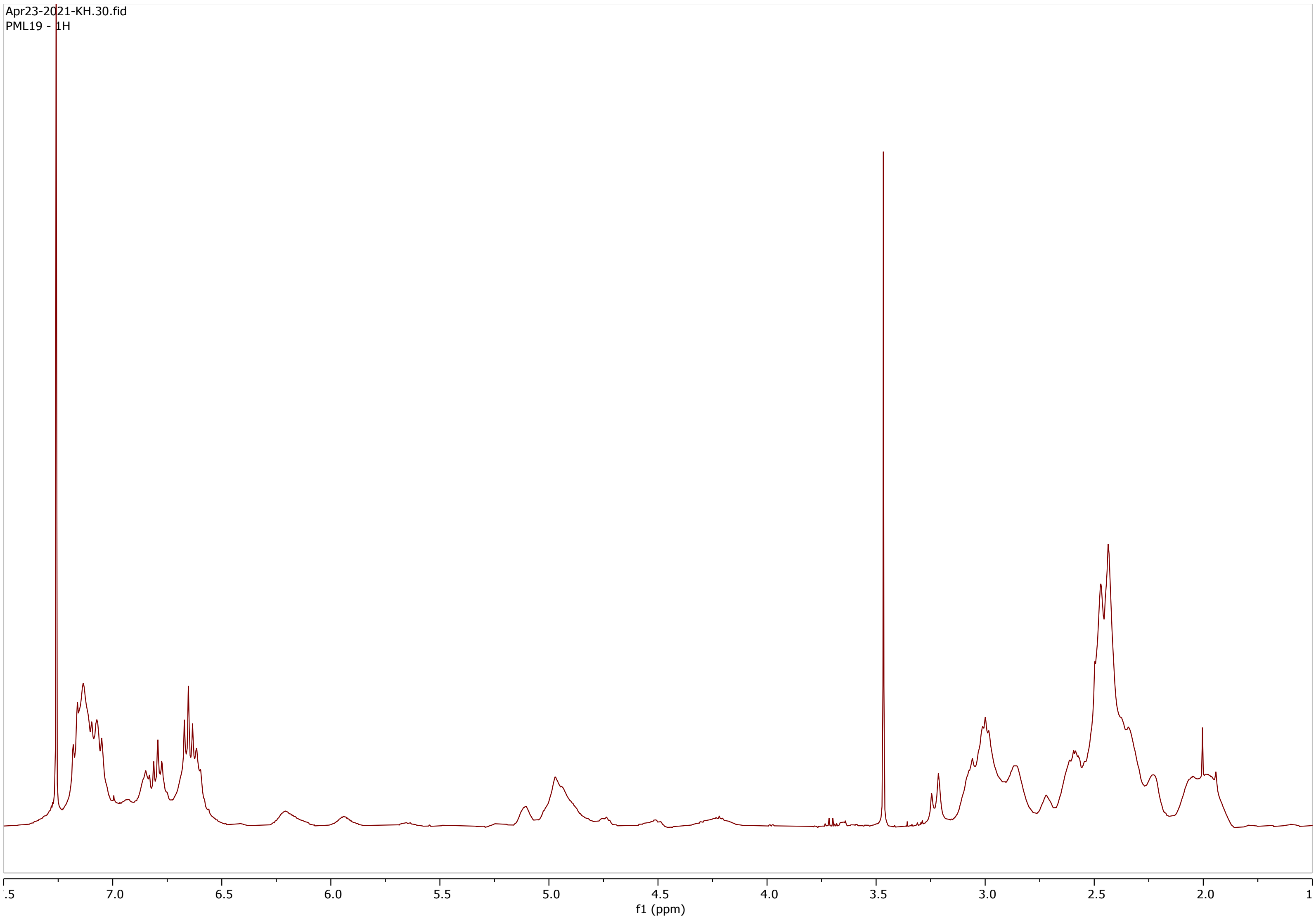

Figure S7. ^{1}H NMR Spectrum of Caledonine (**3**) at rt in $CDCl_3$

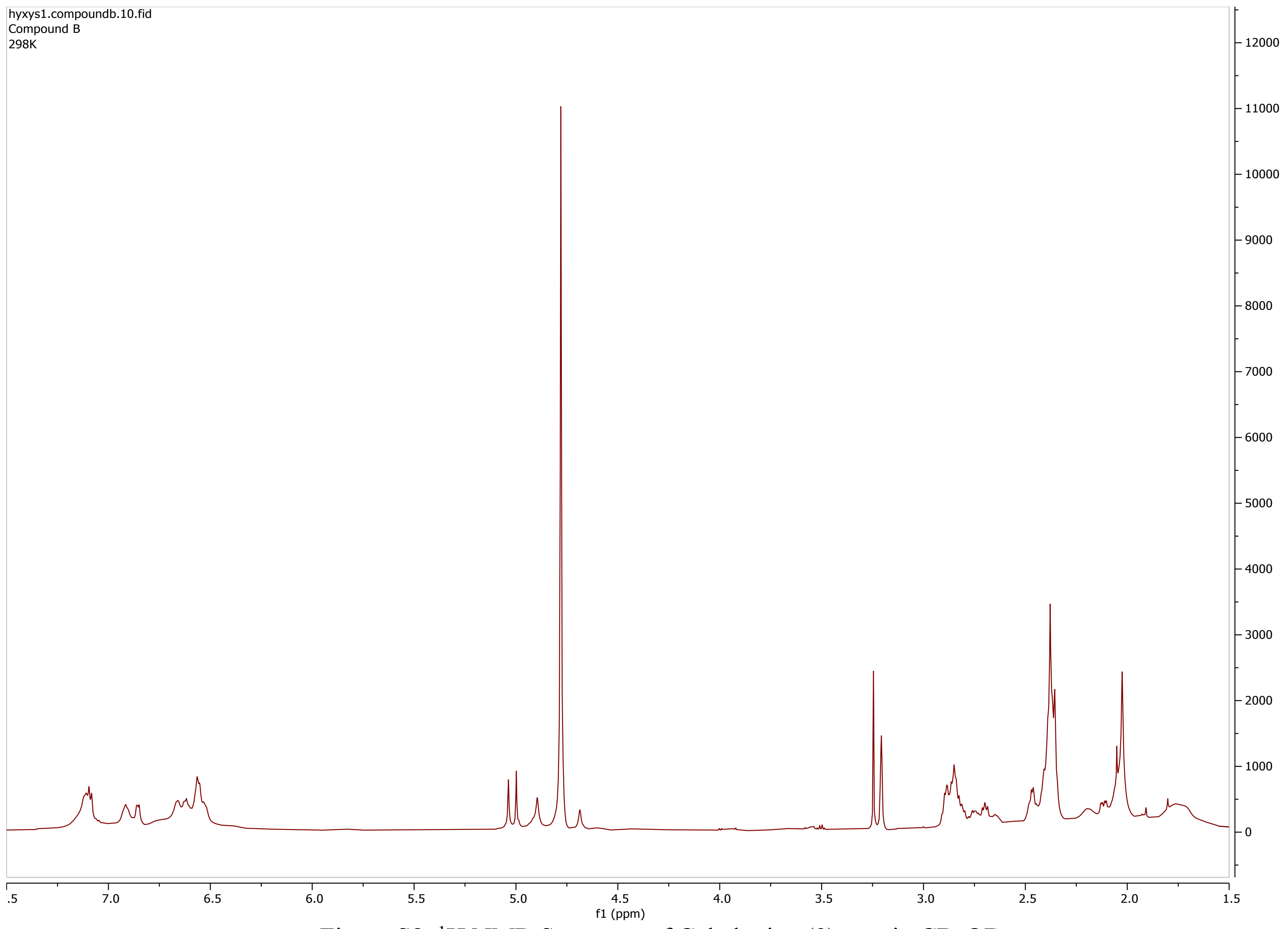

Figure S8. ^{1}H NMR Spectrum of Caledonine (**3**) at rt in CD_3OD

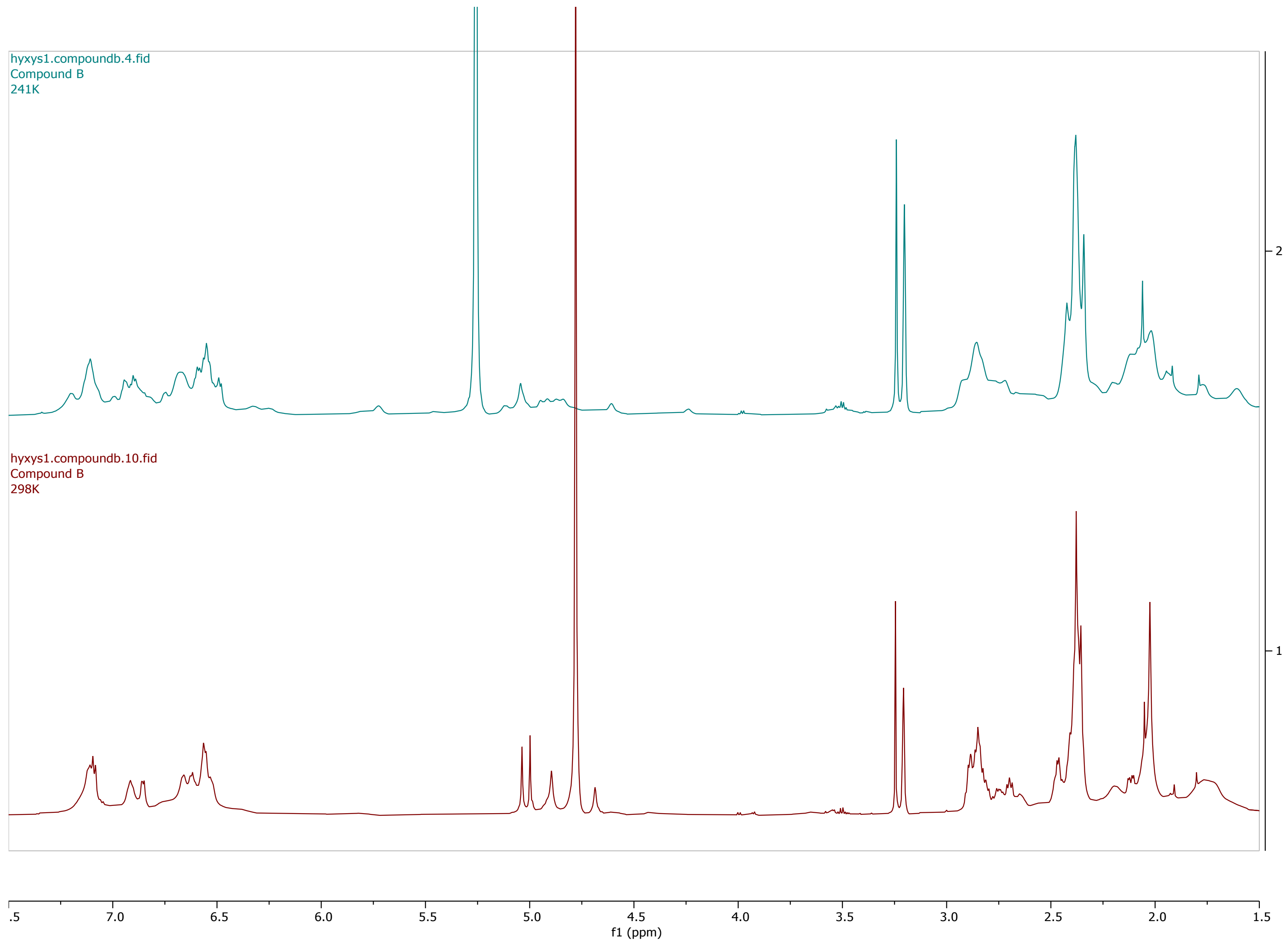

Figure S9. ^{1}H NMR Spectra of Caledonine (**3**) at 241 K and 298 K in CD_3OD

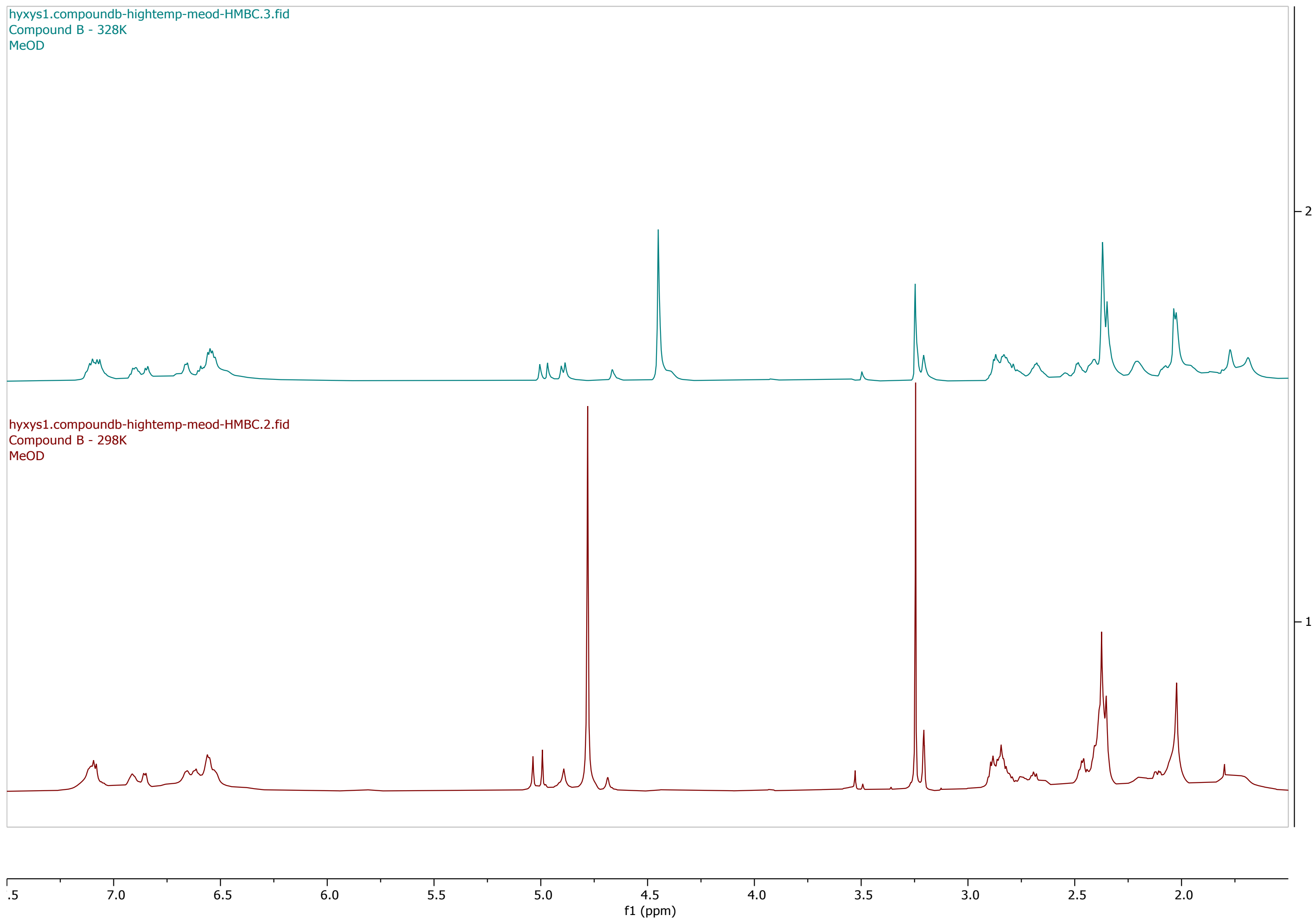

Figure S10. ^{1}H NMR Spectra of Caledonine (**3**) at 328 K and 298 K in CD_3OD

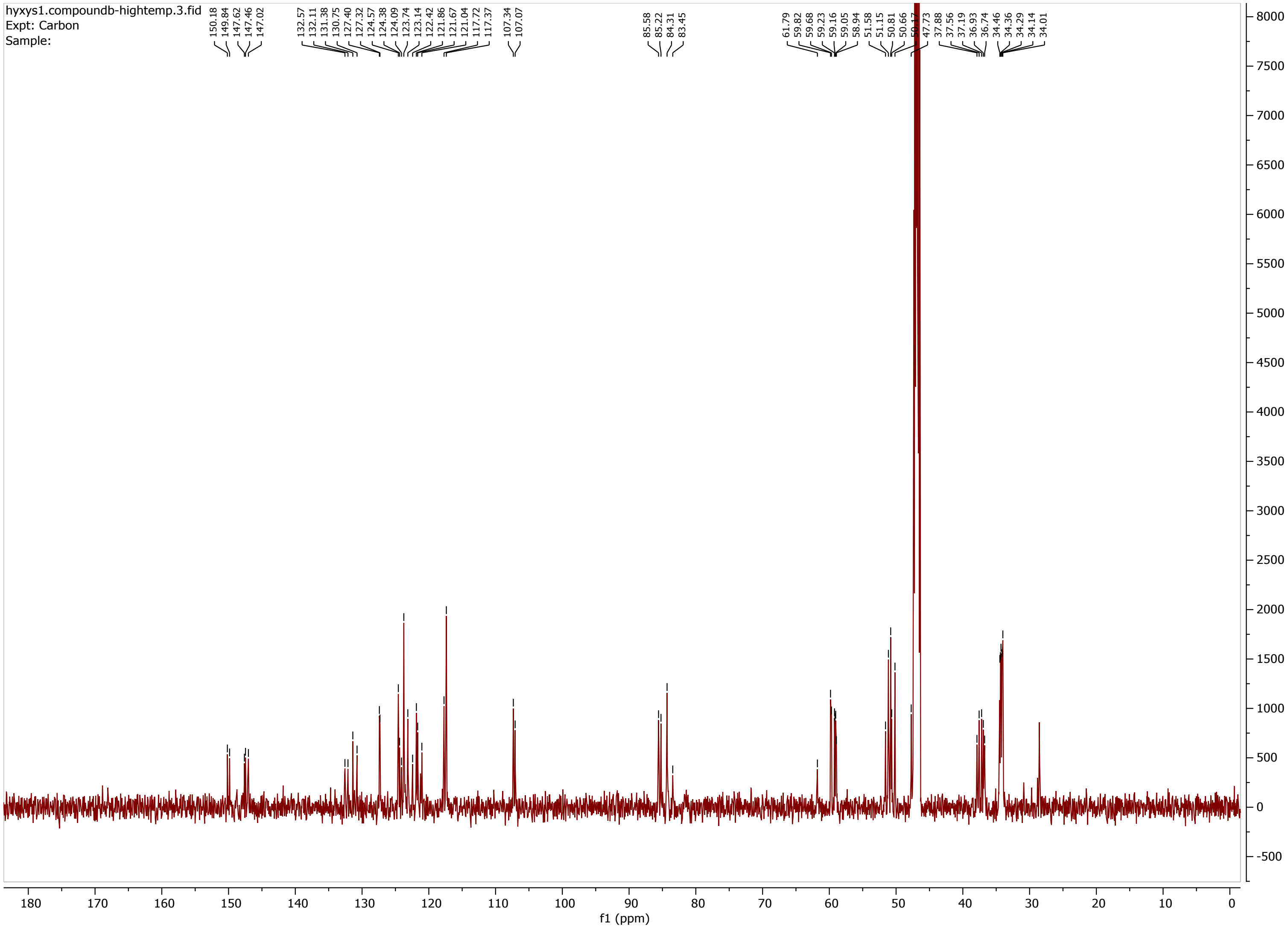

Figure S11. ^{13}C NMR Spectrum of Caledonine (**3**) at 328 K in CD_3OD

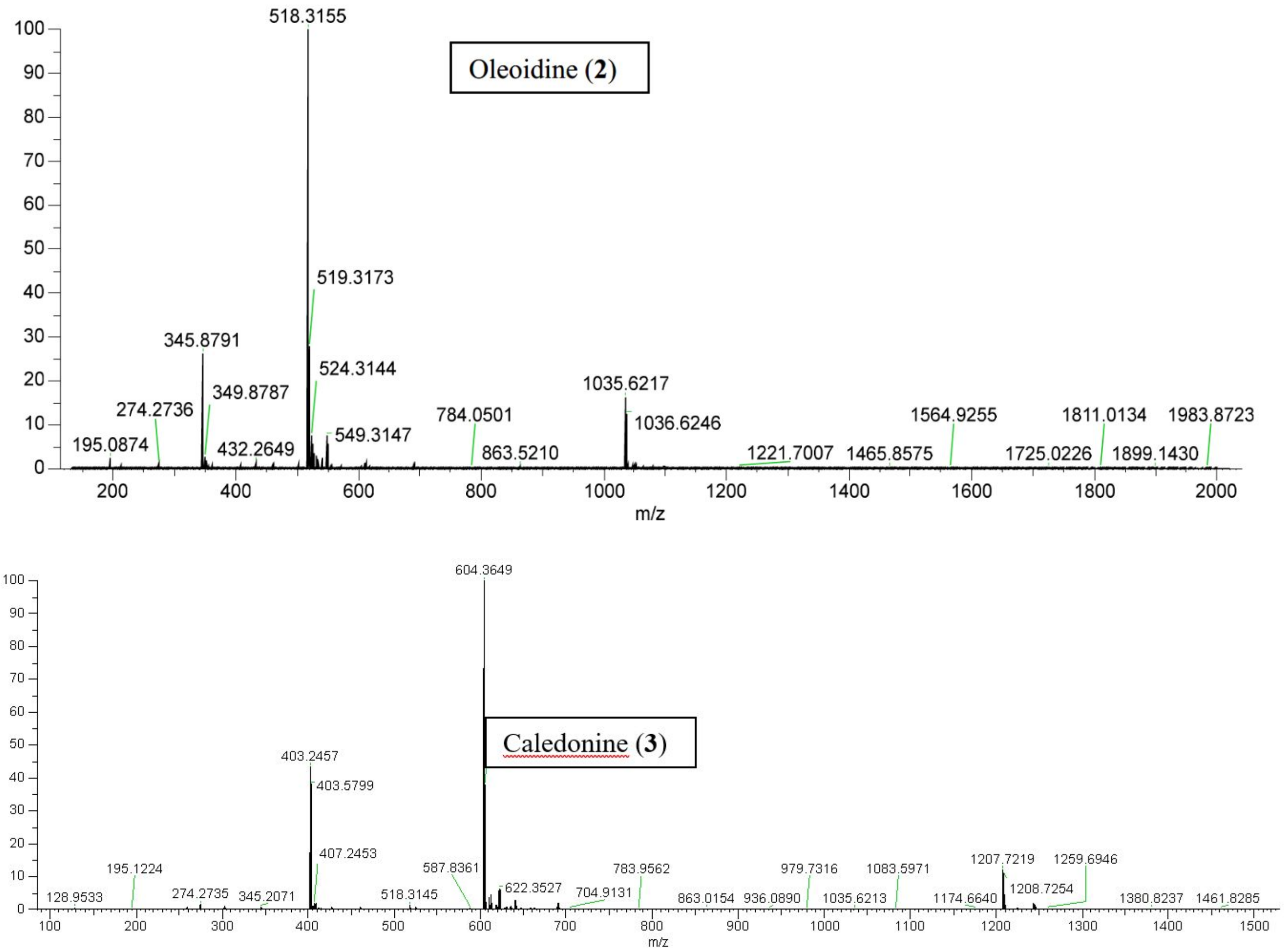

Figure S12. Mass Spectra of Oleoidine (**2**) and Caledonine (**3**)

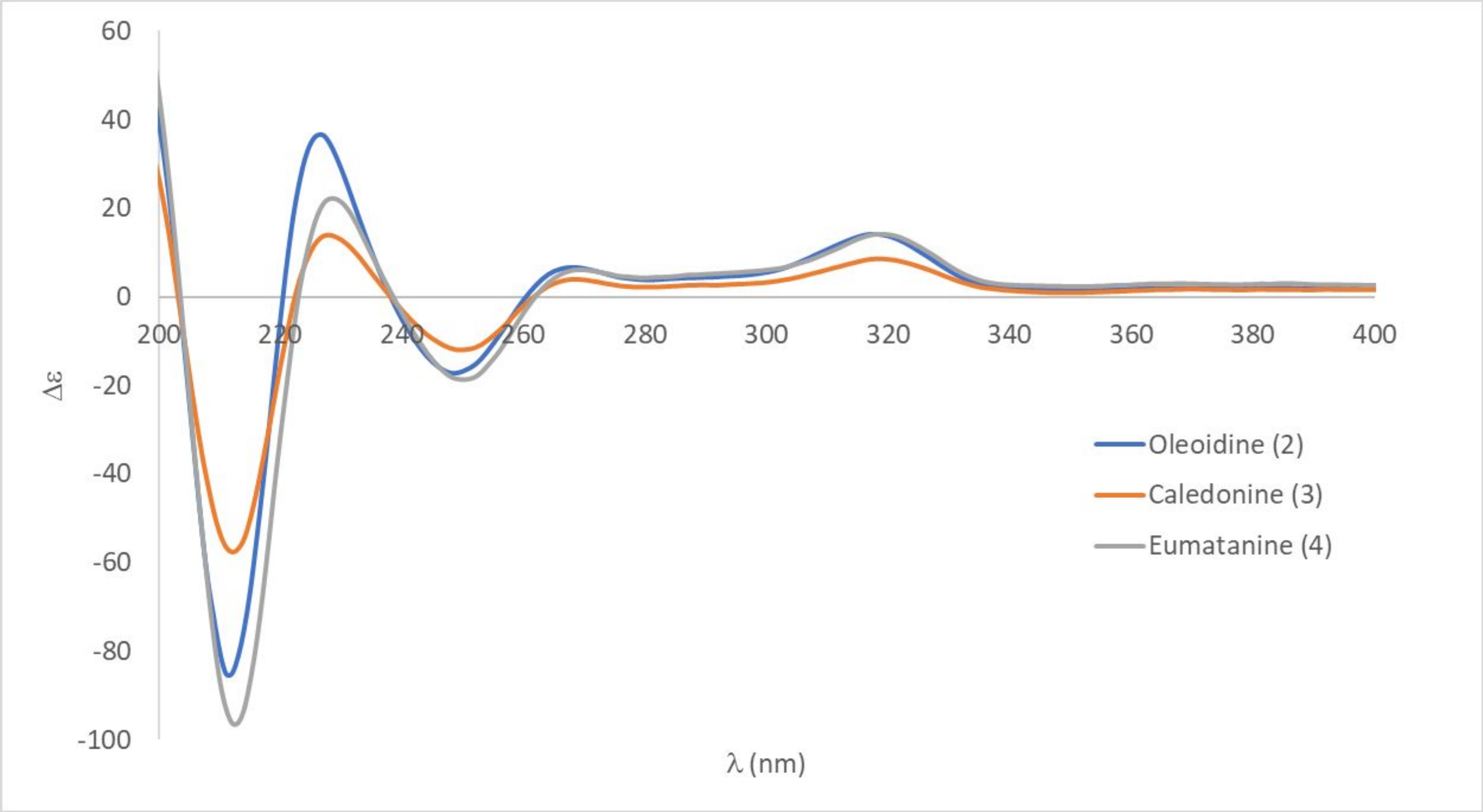

Figure S13. ECD spectra of Oleoidine (**2**), Caledonine (**3**), and Eumatanine (**4**)

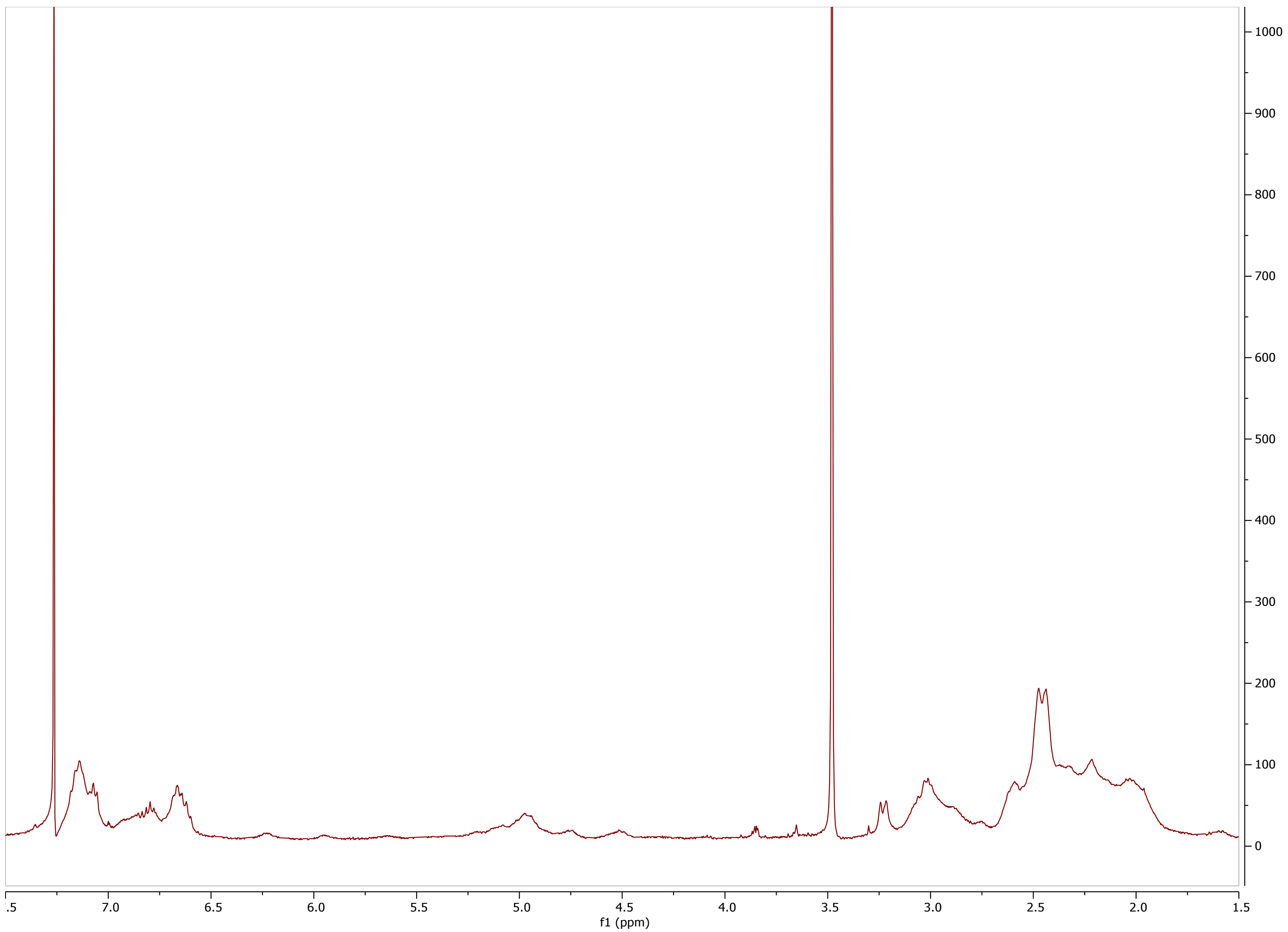

Figure S14. ^{1}H NMR Spectrum of Eumatanine (**4**) at rt in $CDCl_3$

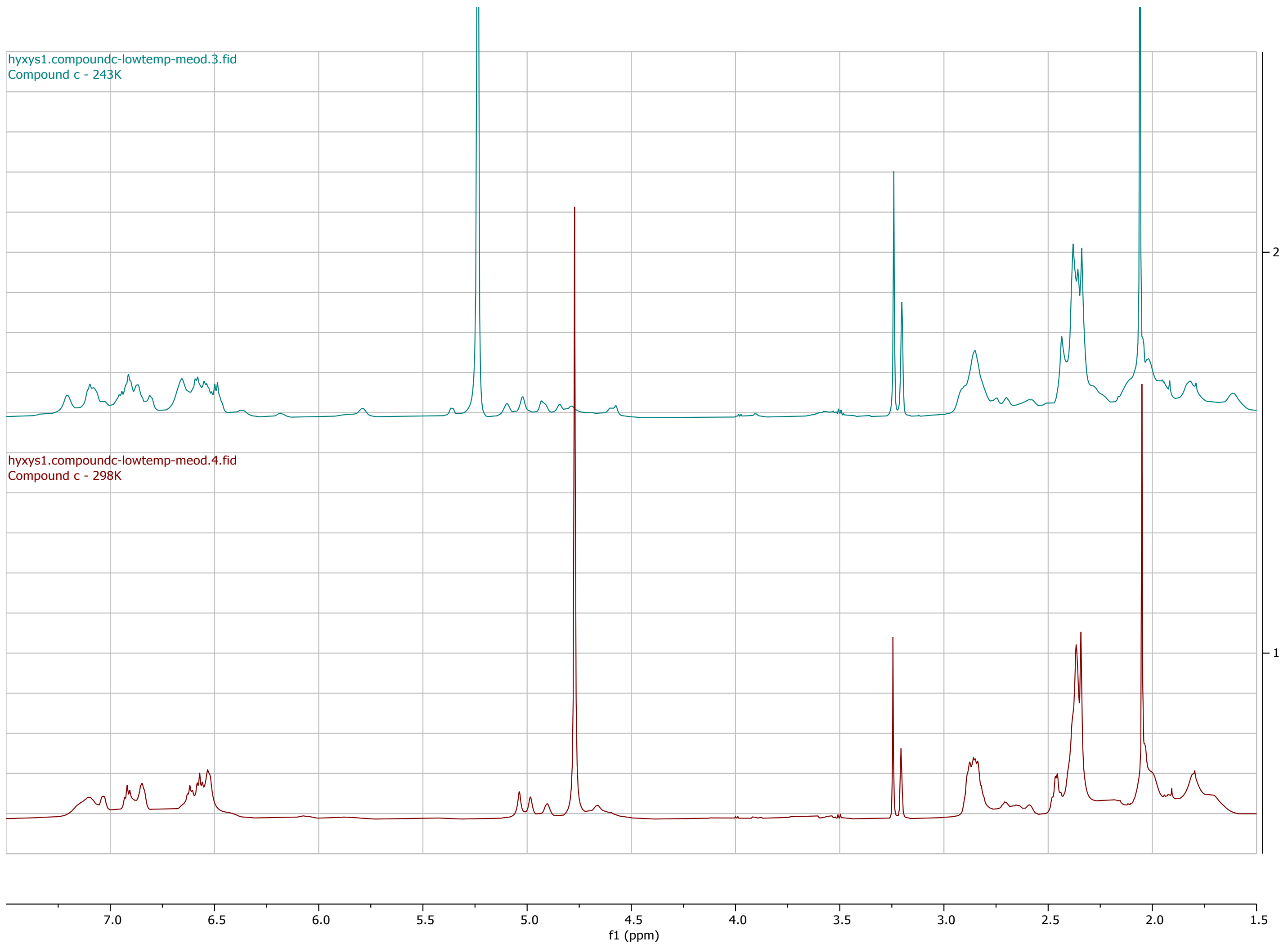

Figure S15. ^{1}H NMR Spectra of Eumatanine **(4)** at 243 K and 298 K in CD_3OD

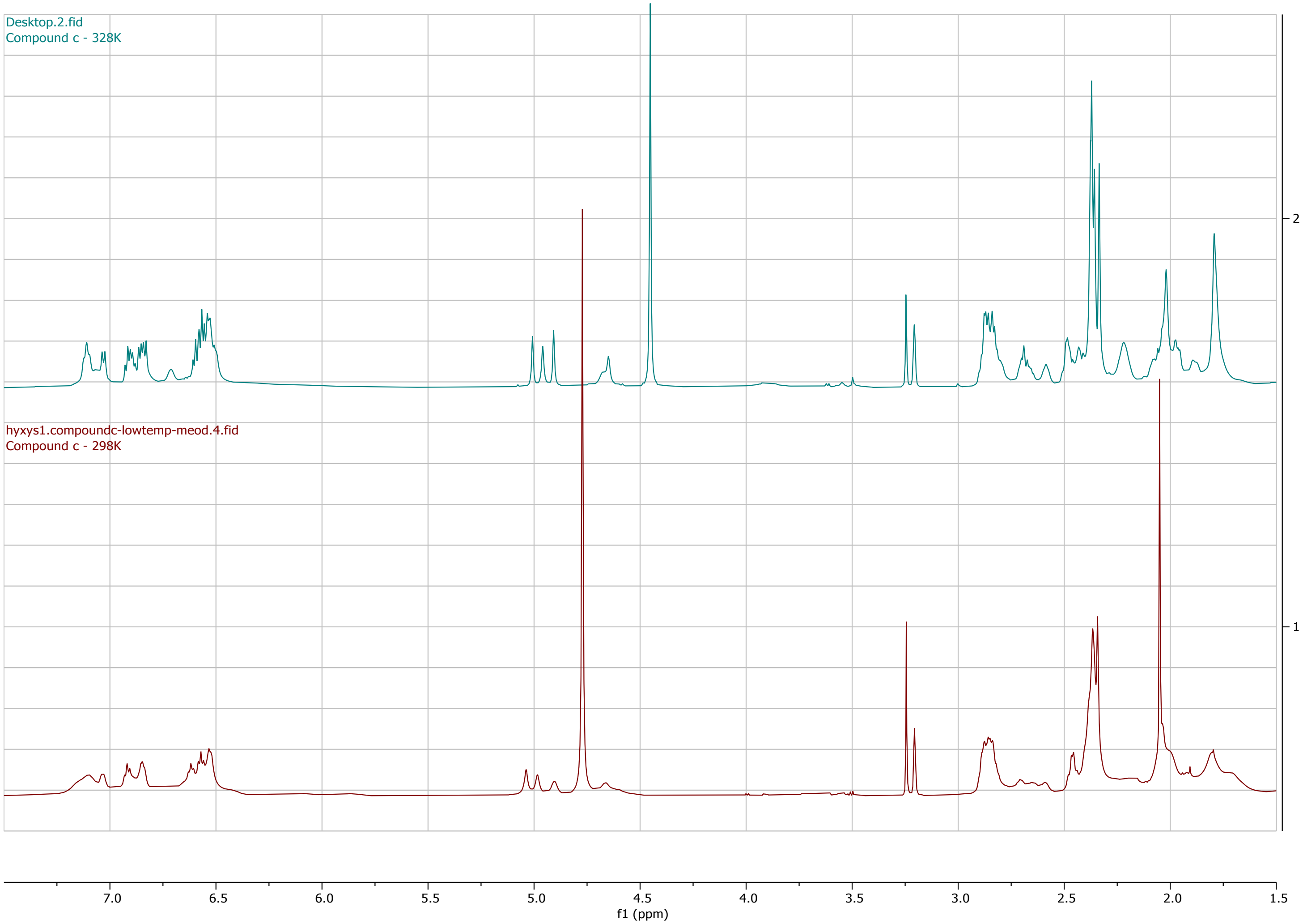

Figure S16. ^{1}H NMR Spectra of Eumatanine **(4)** at 328 K and 298 K in CD_3OD

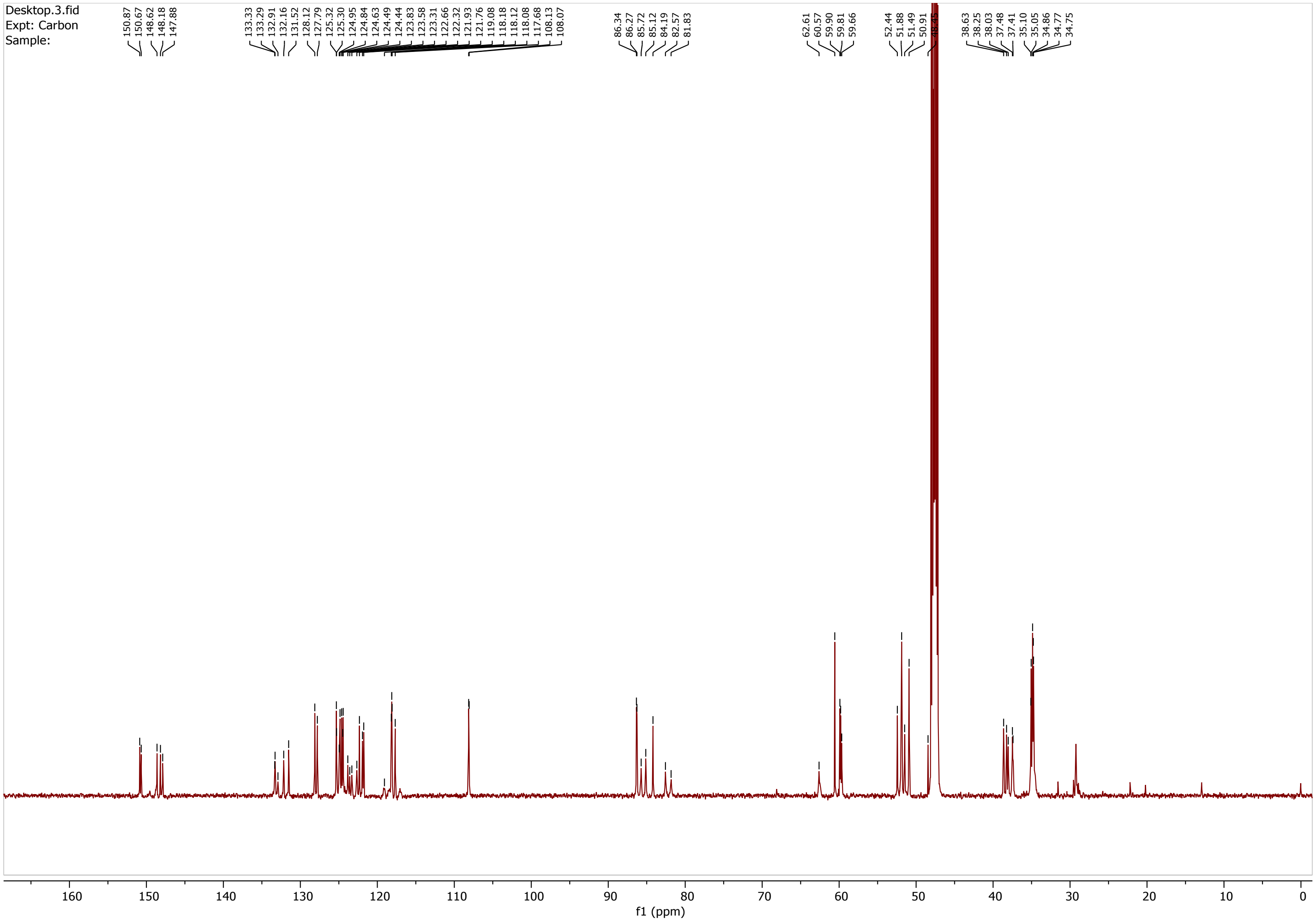

Figure S17. ^{13}C NMR Spectrum of Eumatanine **(4)** at 328 K in CD_3OD

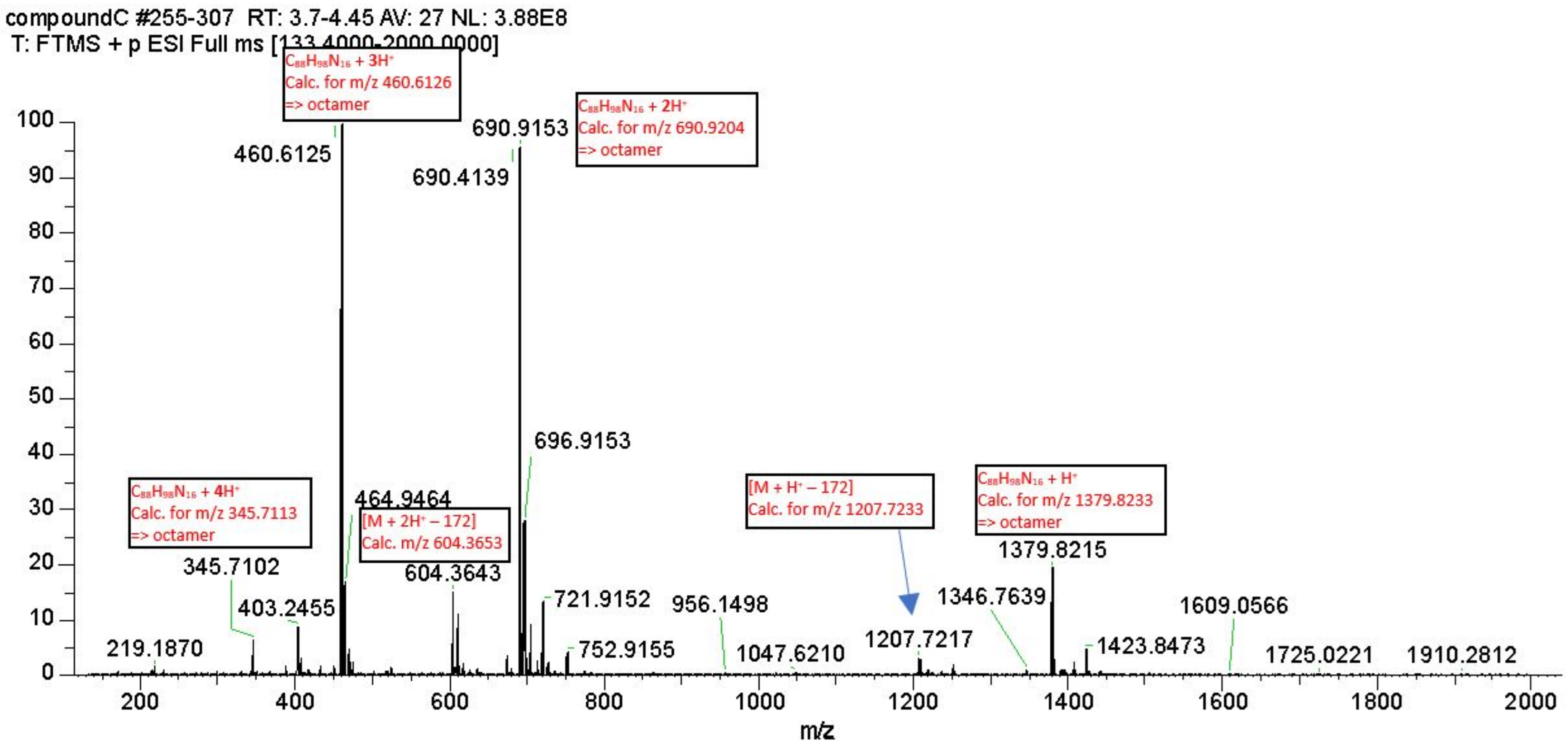

Figure S18. Mass Spectrum of Eumatanine (**4**)

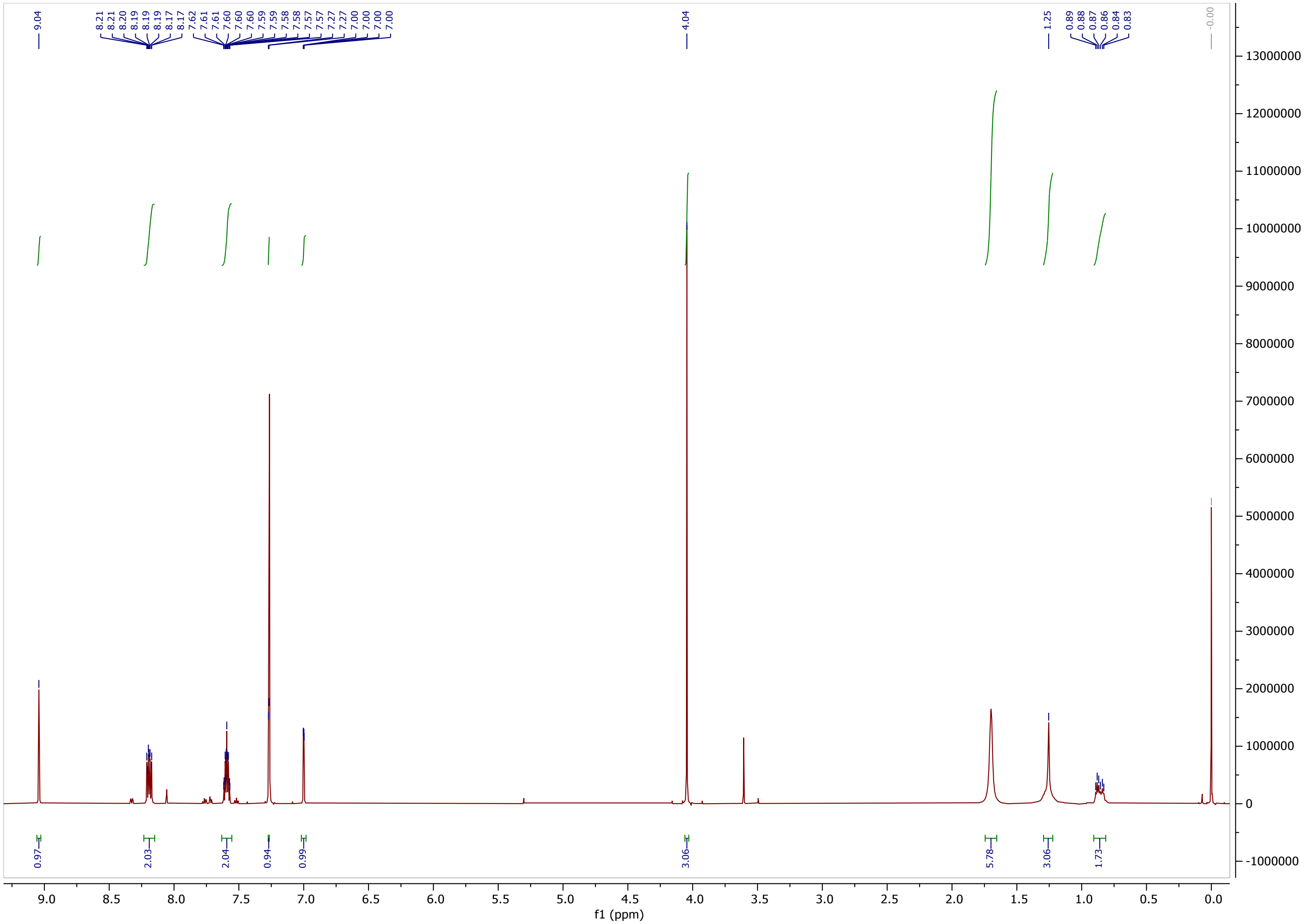

Figure S19. ^{1}H NMR Spectrum of Eumatricine (**5**)

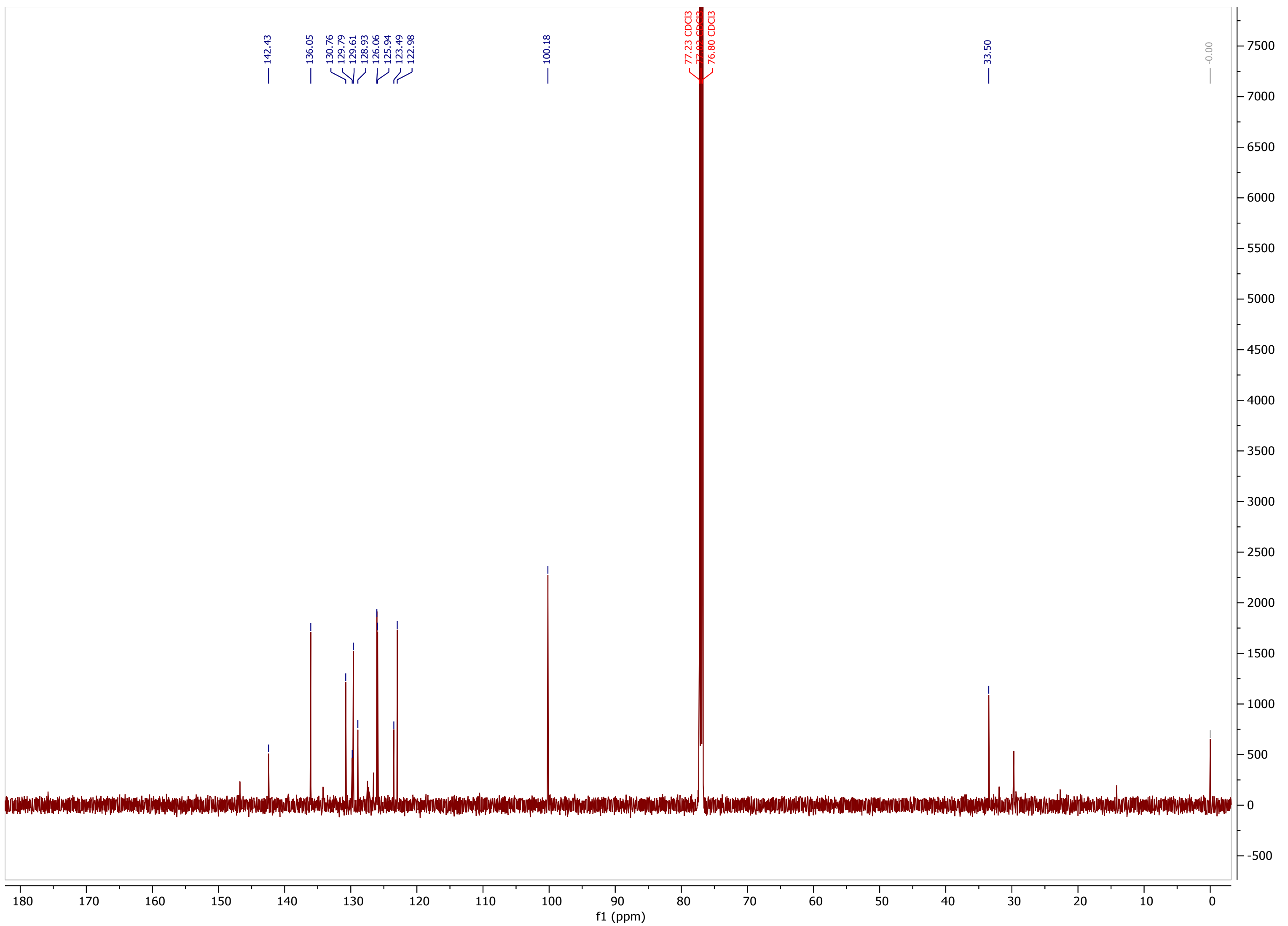

Figure S20. ^{13}C NMR Spectrum of Eumatricine (**5**)

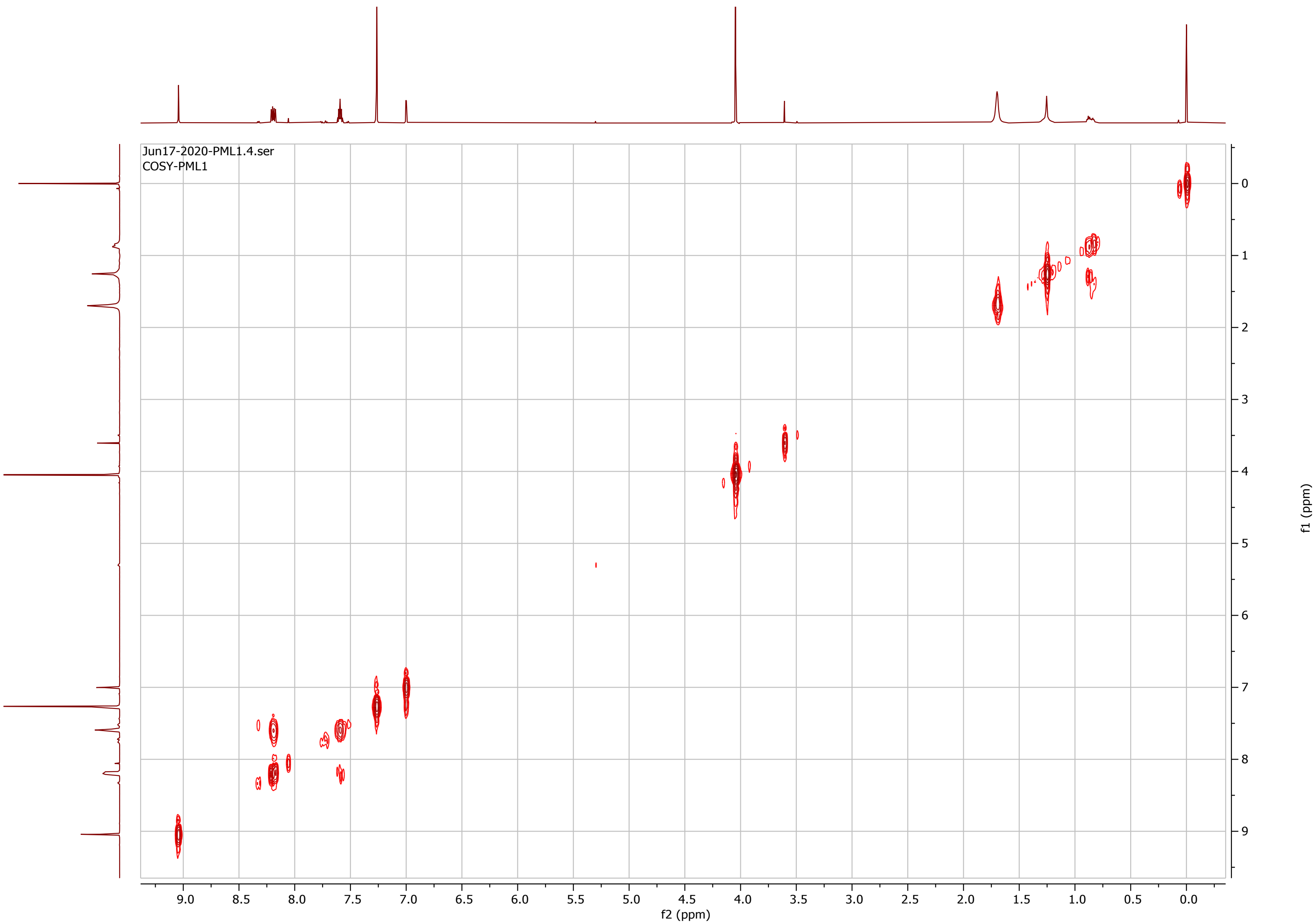

Figure S21. COSY Spectrum of Eumatricine (**5**)

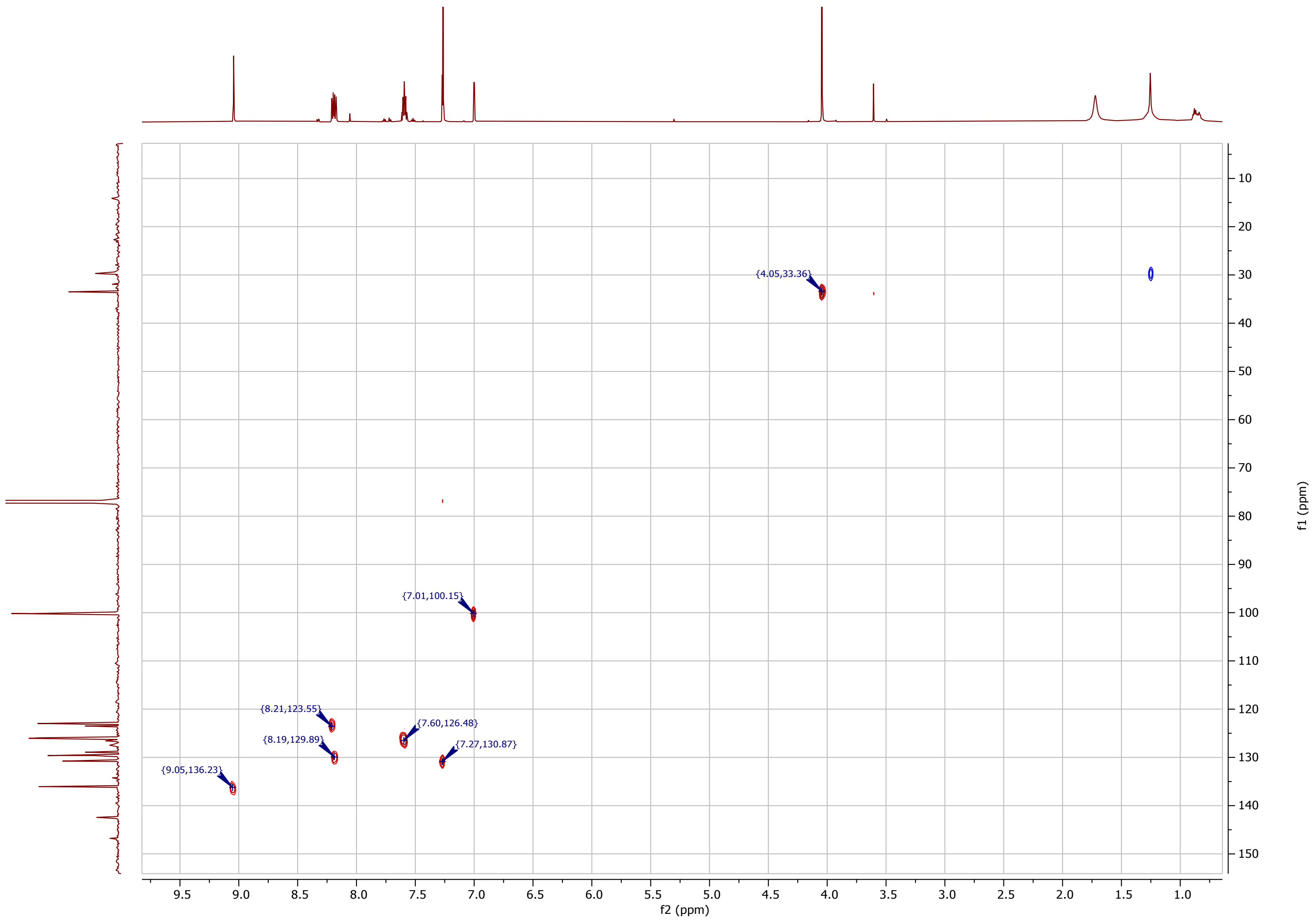

Figure S22. HSQC Spectrum of Eumatricine (**5**)

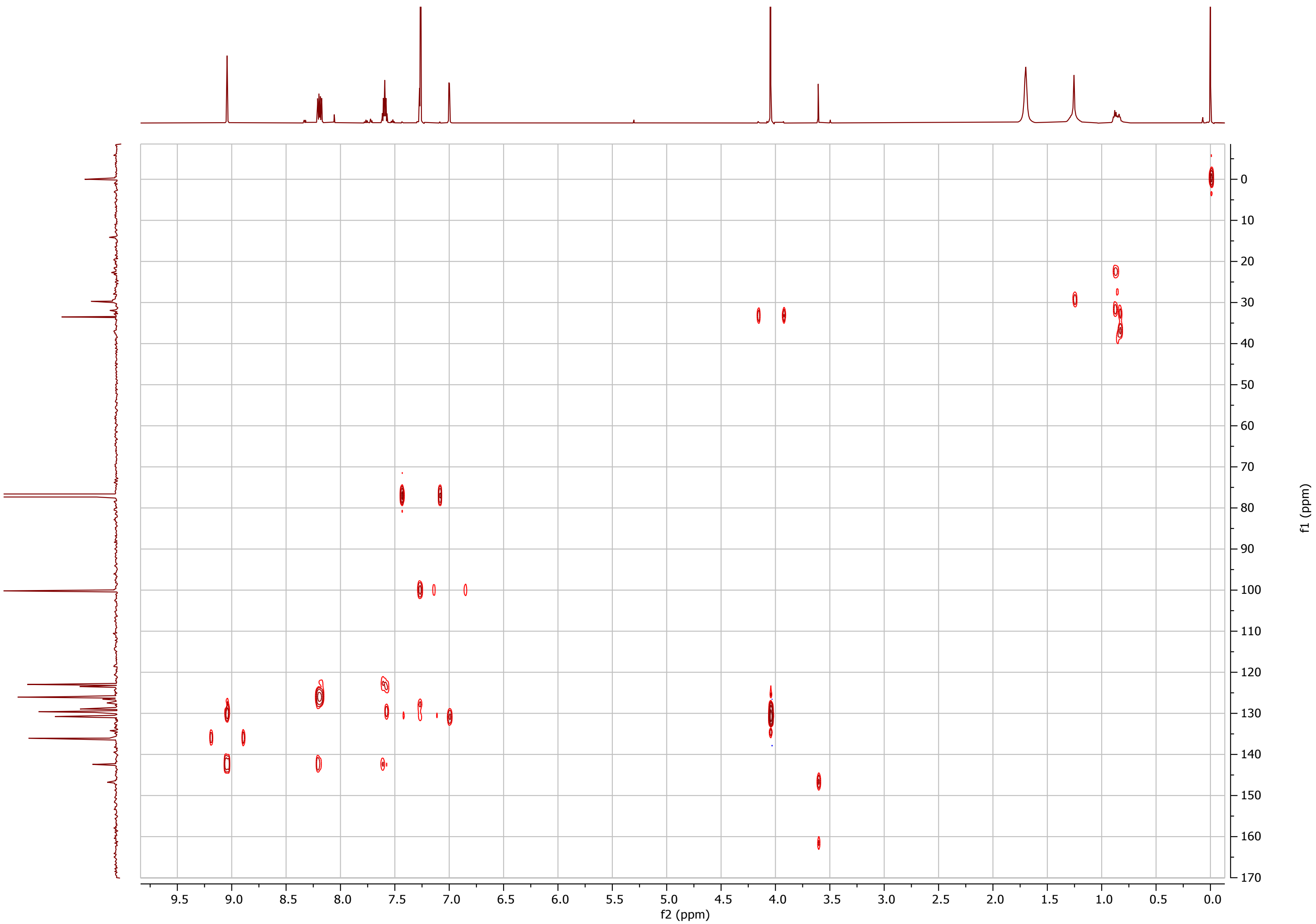

Figure S23. HMBC Spectrum of Eumatricine (**5**)

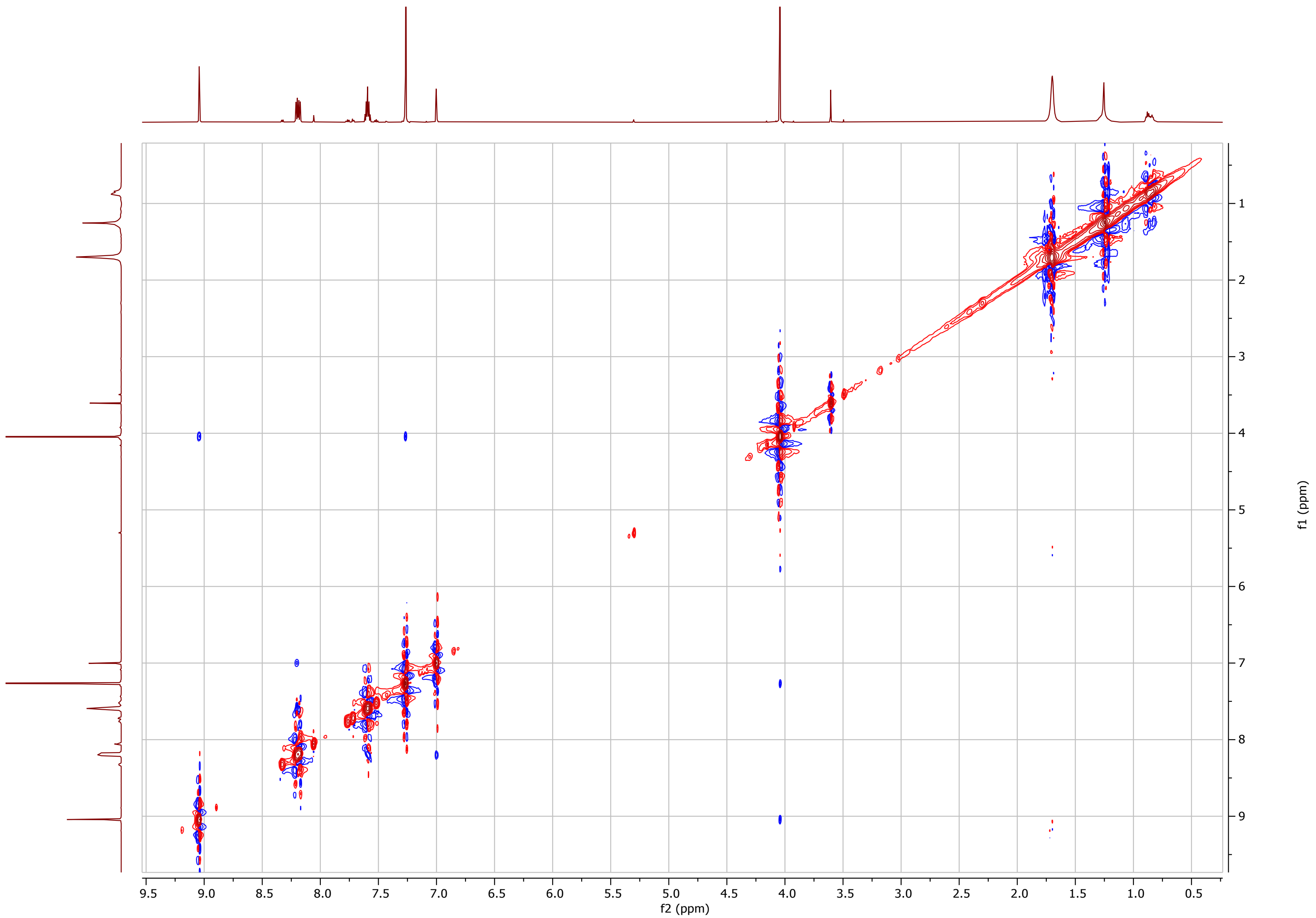

Figure S24. NOESY Spectrum of Eumatricine (**5**)

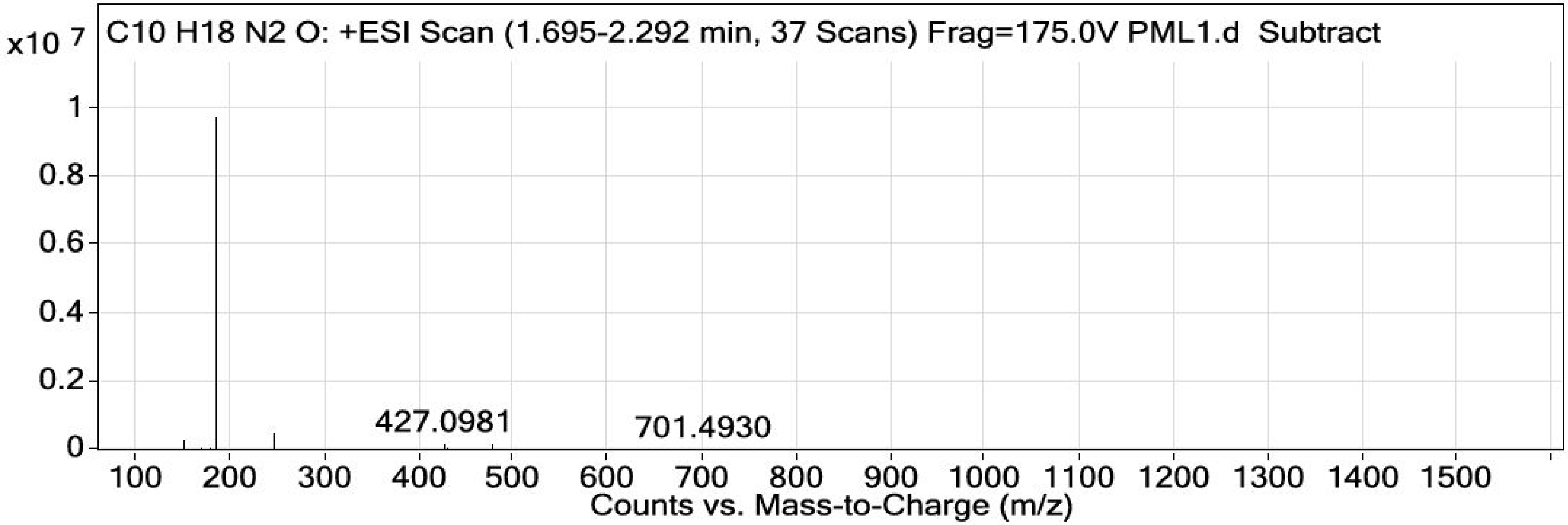

Peak List

m/z	z	Abund	Formula	Ion
149.0237	1	269279		
183.0932	1	9766457	C12 H10 N2	(M+H)+
183.1489		353347.53	C10 H18 N2 O	(M+H)+
184.0956	1	1664578.38	C12 H10 N2	(M+H)+
185.0984	1	87381.25	C12 H10 N2	(M+H)+
245.079	1	512324.81		
246.0819	1	62618.72		
427.0981	1	190984.48		

Figure S25. Mass Spectrum of Eumatricine (**5**)
